# SynLlama: Generating Synthesizable Molecules and Their Analogs with Large Language Models

**DOI:** 10.1021/acscentsci.5c01285

**Published:** 2025-09-17

**Authors:** Kunyang Sun, Dorian Bagni, Joseph M. Cavanagh, Yingze Wang, Jacob M. Sawyer, Bo Zhou, Andrew Gritsevskiy, Oufan Zhang, Teresa Head-Gordon

**Affiliations:** †Kenneth S. Pitzer Theory Center and Department of Chemistry, ‡Department of Bioengineering, and §Department of Chemical and Biomolecular Engineering, 1438 University of California, Berkeley, California 94720, United States; ∥ Department of Chemistry, 5635 University of Minnesota, 207 Pleasant Street SE, Minneapolis, Minnesota 55455, United States; ⊥ Department of Pharmaceutical Sciences, 14681 University of Illinois Chicago, 833 S Wood St, Chicago, Illinois 60612, United States; # Contramont Research, San Francisco, California 94158, United States

## Abstract

Generative machine learning models for exploring chemical space have shown immense promise, but many molecules that they generate are too difficult to synthesize, making them impractical for further investigation or development. In this work, we present a novel approach by fine-tuning Meta’s Llama3 Large Language Models (LLMs) to create SynLlama, which generates full synthetic pathways made of commonly accessible building blocks and robust organic reaction templates. SynLlama explores a large synthesizable space using significantly less data and offers strong performance in both forward and bottom-up synthesis planning compared to other state-of-the-art methods. We find that SynLlama, even without training on external building blocks, can effectively generalize to unseen yet purchasable building blocks, meaning that its reconstruction capabilities extend to a broader synthesizable chemical space than those of the training data. We also demonstrate the use of SynLlama in a pharmaceutical context for synthesis planning of analog molecules and hit expansion leads for proposed inhibitors of target proteins, offering medicinal chemists a valuable tool for discovery.

## Graphical abstract

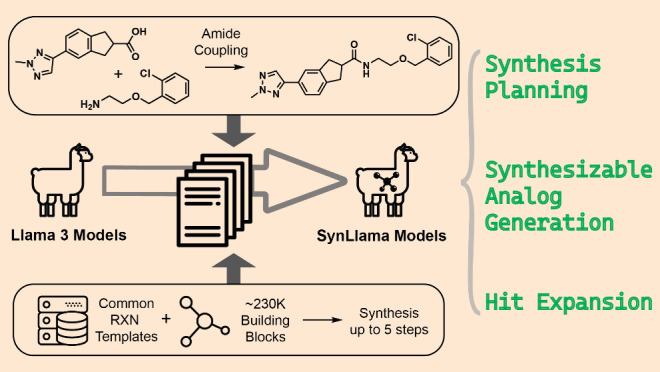

## 1 Introduction

Chemical space is enormous, built up via the exponential rise in functional group combinatorics that define an increasing diverse set of molecules. Traditional approaches that design synthetic pathways of unseen molecules under well-controlled laboratory conditions have been made possible by decades of research in synthetic chemistry, as well as mechanistic studies of key reaction steps and reaction classification.^1,2^ Using the wealth of data accumulated in libraries of chemical reactions, expert systems^3,4^ have been developed that deploy this knowledge to construct multistep pathways to specified end-products. Such methods have become a key tool for the bench chemist, as illustrated by the Chematica software and its follow on commercial product now known as Synthia.^4^

With recent advances in artificial intelligence and deep learning, generative models have begun to contribute to enumerating molecules on the stoichiometric scale. After training on databases containing various small molecules representations,^5–9^ string-based 1D generative models and structure-aware 3D *de novo* methods have paved the way for quick exploration of greater swaths of unseen chemical space.^10–27^ However, even with their exceptional generative capabilities, these models still face one major challenge: their proposed *de novo* molecules lack practical guarantees of synthesizability, which limits their utility in practice.^28–30^ For generative approaches in drug and materials discovery to fulfill their potential, ensuring synthetic feasibility is essential to bridge the gap between *in silico* molecule design and the realistic applicability of computationally generated molecules.

In recognition of this dissonance, efforts have been made to address the problem of the poor synthesizability of *de novo*-generated molecules. One line of research focuses on integrating empirical or deep-learning scoring functions,^31–37^ such as the synthetic accessibility (SA) score^31^ and DeepSA score,^35^ into the objective functions of learning algorithms. However, optimizing only the synthesizability score can still lead to the generation of unsynthesizable molecules, because the scoring functions rely on identifying common fragments or reactive centers in molecules.^38^ In addition, they often assign bad scores to complex yet synthesizable molecules that require multistep synthetic pathways, causing generative models to miss viable candidates.^39^ Others have proposed improving synthesizability by building molecules from common molecular fragments,^40–43^ but they still do not guarantee synthesis as these methods do not explicitly consider the reaction pathways to build the molecular candidates. Another line of research integrates the explicit use of computer-assisted synthetic planning (CASP) software^44,45^ into the optimization.^46^ However, the computational overhead of these methods is significant and the quality of the optimized molecules can be variable.^47^

Alternatively, proposing synthesizable molecular candidates using commercially available building blocks and commonly known organic reaction templates^44,48^ offers better synthetic tractability over simple molecule scoring. Importantly, this strategy is appealing to bench and medicinal chemists since it offers actionable synthesis pathways for them to examine, refine, and execute. Some recent models in this direction apply rule-based synthesis and optimization on building blocks or entire synthetic pathways to generate novel molecules with desired chemical properties.^4,49–53^ Other models condition on input molecules to propose synthetic pathways using commercially available building blocks and well-validated reaction templates for either full construction of the target molecule or the generation of structurally similar analogs in a forward synthesis manner within the predefined chemical search space.^54–56^ For example, SynNet^54^ constructs synthetic trees via Markov Decision Processes (MDPs) and uses multilayer perceptrons to choose the next action space. More recent models such as ChemProjector^55^ and Synformer^56^ use transformers to decode for the next action space and have achieved good empirical performances for target and analog molecule reconstruction.

A compelling alternative is the use of Large Language Models (LLMs) due to their foundational nature and adaptability to downstream tasks.^57^ LLMs inherently possess extensive chemical knowledge, and recent advancements have focused on extracting and applying this knowledge for predictive and optimization tasks using natural language guidance.^58–61^ Furthermore, after fine-tuning, LLMs can perform as good or better than chemical language models trained solely on chemical representations, all while requiring less data.^27^ The efficiency and unexpected performance gains from fine-tuning LLMs thus motivate us to explore their potential in more complex tasks, such as synthesis planning, which could pave the way for new chemical discoveries.

Herein, we present SynLlama, an LLM-based tool built on the open-source Llama-3.1-8B and Llama-3.2-1B foundation models^62^ to deduce synthetic routes for target molecules or structurally related analogs. Specifically, the LLM component of SynLlama operates as a constrained retrosynthesis module that breaks input molecules into building blocks (BBs) via well-validated (RXN) sequences, and the reconstruction module searches commercially available BBs based on LLM predictions and builds up molecules within a diverse yet synthesizable chemical space. As an illustration of utility, SynLlama demonstrates competitive performance in key tasks for drug discovery, including synthesis planning for target and analog molecules of pharmaceutical interest and expansion around existing molecular drug hits and leads. Moreover, because of its generative nature, the LLM component of SynLlama has the added ability to explore commercially available building blocks beyond the predefined synthetic space introduced during training, an ability that previous models lack. By integrating molecular design with synthetic feasibility, SynLlama represents a step forward in bridging computational chemistry with synthetic chemistry, providing chemists with actionable and experimentally accessible molecular candidates.

## 2 Methods

The SynLlama workflow, illustrated in Figure 1, is designed to generate synthesizable compounds within an expanded chemical space. When an input molecule passes through this workflow, it can either be fully reconstructed through valid synthetic pathways or the workflow will produce a structurally similar yet synthesizable analog along with its synthesis route. To transform general-purpose LLMs, like the Llama 3 models,^62^ into expert models for synthetic pathways, we use three key components: 1) a reliable and diverse set of reaction data that covers a large synthesizable chemical space, 2) an efficient supervised fine-tuning (SFT) strategy to train a general-purpose LLM on these reaction data, and 3) a reconstruction algorithm that can convert the output of the fine-tuned LLM into valid synthesis routes, ensuring the proposed molecules lie within a commercially available chemical search space. These components are crucial for leveraging LLMs, which are known to perform well in diverse chemistry tasks,^63,64^ to specialize in synthetic modeling.

**Figure 1:**
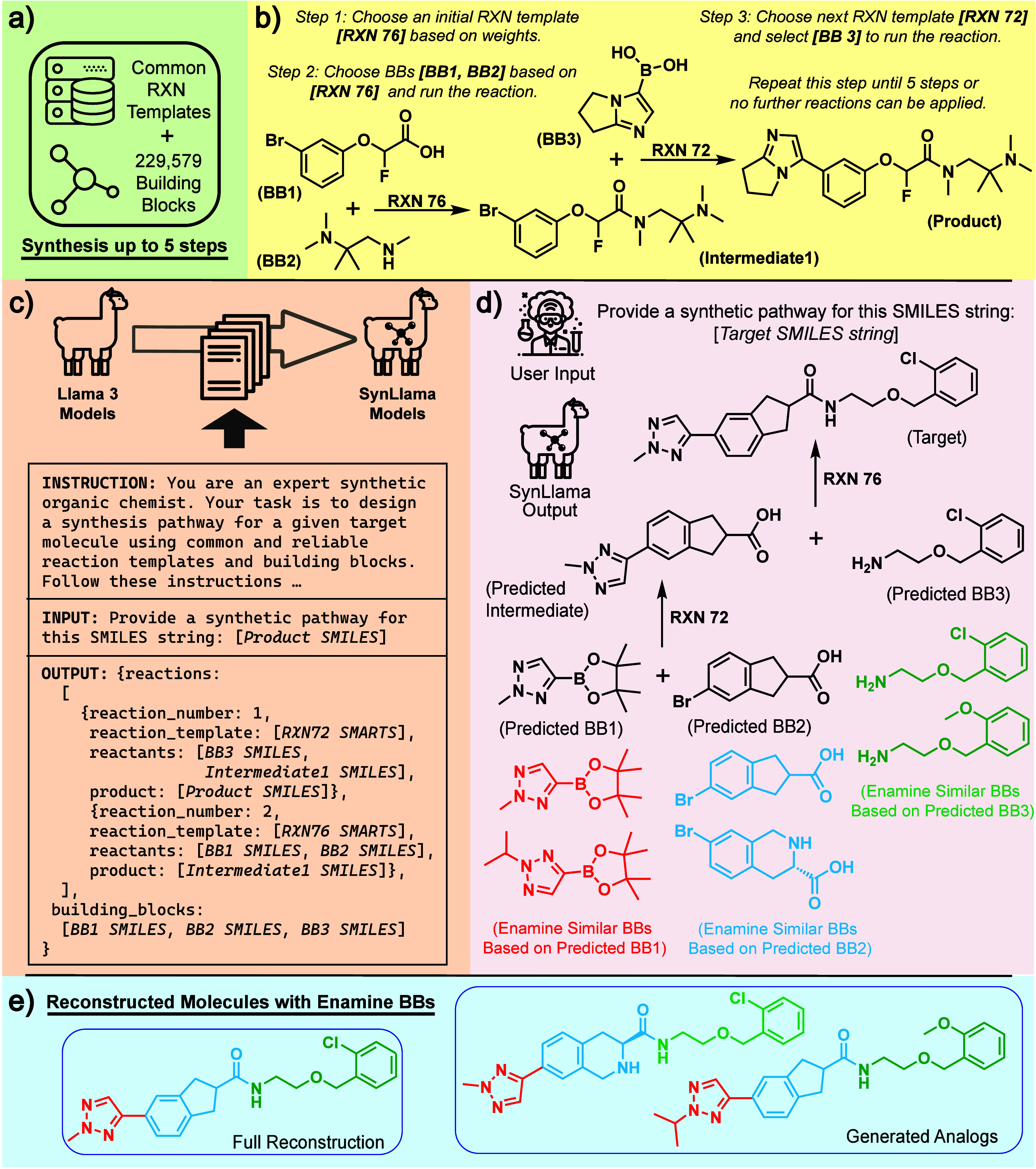
**Overview of the SynLlama workflow including data generation, supervised fine-tuning, inference, and reconstruction.** (a) The predefined synthesizable chemical space of reaction templates (RXN) and building blocks (BBs) covers billions of molecules. (b) An example of synthesis data and its generation process from the defined synthesizable chemical space to create training examples. Here, RXN 76 represents amide coupling and RXN 72 represents Suzuki coupling. (c) A schematic representation of supervised fine-tuning that converts Llama 3 models to SynLlama models, along with the instruction, input, and output for the example synthesis in (b). (d) SynLlama’s inference on an unseen test molecule. Black represents SynLlama’s raw retrosynthetic output consisting of RXN sequences and predicted BBs, while colored BBs indicate the top two most similar BBs to the predicted ones from the Enamine building block library. Here, RXN 76 represents amide coupling and RXN 72 represents Suzuki coupling. (e) Reconstructed molecules were constructed using the predicted reaction sequences and similar building blocks from the Enamine building block library. In this example, all predicted building blocks are present in the Enamine library, allowing for the complete reconstruction of the input molecule and the generation of close analogs.

### 2.1 Reaction Data for Training and Testing Sets

As illustrated in Figure 1(a), our defined chemical space for training consists of molecules that can be synthesized in at most five steps with Enamine building blocks^9^ (BBs) and 2 sets of well-validated common organic reactions (RXNs). To define the training and testing BB data, we apply a time split whereby all Enamine BBs from the August 2024 release serve as the training BBs, and all new BBs from their February 2025 release that were not in the training set comprise the testing BBs. This procedure results in ∼230,000 BBs for training and ∼13,000 BBs for testing. Later in Section 3.1 we consider the target reconstruction for unseen molecules which are taken from the Enamine Diversity Set^9^ and ChEMBL data set.^8^

We define two sets of reaction templates (RXN) that operate on the Enamine BBs. RXN 1 is formulated as a set of 91 reaction templates selected by Gao et al.^54^ from the works of Hartenfeller et al.^65^ and Button et al.^66^ RXN 2 is comprised of 115 reactions selected by Gao et al.^56^ that contains reactions used to create the Enamine REAL space^9^ plus some reactions from RXN 1. All reaction templates are accessible to both sets of BBs, thus defining the training and testing chemical spaces. As a result, there are ∼10^30^ molecules within this space that can be represented by a synthesis path that comprises a sequence of BBs and RXNs.

To enumerate molecules within this space, we used an iterative approach by selecting RXN templates and searching for compatible BBs. Specifically, as demonstrated in Figure 1(b), the selection of the initial RXN is guided by a probabilistic model based on the number of compatible BBs. Within these compatible BB reactants, the initial BBs are selected at random to form an intermediate via the selected RXN template. This intermediate is then used to match for subsequent RXNs and recruits additional BBs to expand the molecular synthesis pathway until no further reactions are possible, or the reaction reaches five steps.

After training on these representations, the resulting LLM will be able to build powerful connections by mapping input molecules to a sequence of BBs and RXNs that create linear synthesis routes for the testing sets of molecules. To examine the model's generalizability to hard cases of synthesis, we also construct test sets of branch synthesis in which molecules have at least one or more reaction steps involving intermediates as reactants. When constructing the branching synthesis test sets using both RXN 1 and 2 templates, we keep track of two synthesis trees at a time and check whether the intermediate molecules from both synthesis trees can react further with at least one reaction template from the corresponding RXN. We then filter for molecules that have at least one reaction step, with reactants being intermediates to create the two test sets.

### 2.2 Supervised Fine-Tuning and Inference from SynLlama

To create the SynLlama model, we need to establish data generation protocols for supervised fine-tuning (SFT) of the Llama 3 models, as schematically shown in Figure 1(c). When generating reaction data in text format, we choose to represent the BBs and intermediates along the synthetic pathway using SMILES^67^ strings, while RXNs are explicitly defined in the SMARTS^68^ format. These structured chemical notations are designed to enhance SynLlama’s ability to systematically identify and deconstruct bonds according to RXN templates, effectively dismantling input molecules into building-block-sized fragments.

Since our goal is for SynLlama to learn to link molecules with their synthesis routes, our prompt-response pairs are structured according to retrosynthesis, as depicted in Figure 1(c) and shown in detail in Supplementary Figure S1. Such engineered prompts and responses allow the SynLlama model to learn to construct synthesis pathways for the input molecules by inferring sequences of BBs and RXNs as well as the intermediate steps. While theoretically the model could predict BBs and RXNs without intermediates, we still include them in individual reaction steps in the hope of activating the inherent chemical knowledge in LLMs and enhancing their understanding of synthesis patterns. We have included some additional design choice analysis of forward synthesis versus retrosynthesis and whether to apply a drug-like product molecule filtering in the Supporting Information.

We have considered both Llama-3.1-8B (8 Billion parameters) and Llama-3.2-1B (1 Billion parameters) for SFT using data sets of varying sizes. Specifically, Llama-3.1-8B is fine-tuned with data sets containing 100k and 500k synthesis routes, requiring 40 and 240 A40-GPU hours, respectively. Llama-3.2-1B, on the other hand, is trained with data sets containing 500k and 2M synthesis entries, requiring approximately 60 and 240 A40-GPU hours, respectively. Herein, we refer to the trained models as SynLlama (parameter count)-(number of reactions trained) in the first part of the Results. For example, SynLlama-1B-2M represents a model fine-tuned from Llama-3.2-1B with 2M synthesis routes. Further details of the SFT are provided in the Supporting Information.

After training the SynLlama models, we applied the consistent prompt setup to perform inferences on molecules. For any given molecule, the SynLlama models predict reaction sequences in SMARTS format and generate SMILES strings for all of the reactants, products, and BBs for the reactions they predict. During inference time, the instruction to SynLlama remains the same, and SMILES strings in the input section are substituted with strings specified by the user. As depicted in Supplementary Figure S1, the responses of the SynLlama models follow the output structures enforced by the prepared training prompt-response pairs. To be more specific, the output response section consists of two parts: reactions and building blocks. In the “reactions” component, the model sequentially deconstructs the target molecule by breaking bonds using provided reaction templates in a retrosynthetic manner. At each step, it predicts a reaction template along with the reactants and product of the reaction, continuing until no further reactions are possible. Then, in the “building blocks” section, the model compiles all building blocks, namely, reactants from each reaction that are not products in other reactions, identified from the “reaction” section. A visual representation of the inference process is illustrated in black ink in Figure 1(d).

### 2.3 SynLlama Model Benchmarks

Since we are formulating the synthetic tasks using purely language-based modeling, where all reactions are expressed in SMARTS templates and molecules in SMILES strings, it is important to quantify the capacity of SynLlama for instruction following and comprehension of reaction chemistry. To assess SynLlama’s ability to follow instructions, we select three benchmarking criteria as shown in Table 1. The first is “Valid JSON”, which examines whether the output format is a parsable JSON following the fine-tuned templates that will be necessary for the downstream reconstruction algorithm. The second criterion is “Template Memorization”, which assesses the model’s ability to memorize the provided reaction templates that define our synthesizable chemical space. Lastly, we benchmark on “BB Selection”, which evaluates whether the “building blocks” section in the responses can accurately identify and select all of the building blocks from the “reactions” section of the responses.

**Table 1:** Benchmarks of SynLlama Inferences on 1000 Training, Testing, and ChEMBL Data^a^

		Model Config			
Data set	Category	8B-100k	8B-500k	1B-500k	1B-2M
Training Data	Valid JSON	96.20%	97.20%	96.60%	98.00%
	Template Mem.	99.95%	100.0%	100.0%	100.0%
	BB Selection	99.80%	100.0%	99.72%	99.96%
	Valid SMILES	94.74%	99.53%	95.17%	99.46%
	Matched Reactants	78.62%	96.42%	80.19%	97.64%
	Good Products	78.34%	96.58%	81.26%	98.58%
					
Testing Data	Valid JSON	91.00%	94.20%	88.70%	93.90%
	Template Mem.	99.91%	100.0%	99.97%	100.0%
	BB Selection	99.75%	99.96%	99.73%	99.98%
	Valid SMILES	94.37%	99.13%	87.66%	99.50%
	Matched Reactants	77.51%	94.83%	65.63%	96.90%
	Good Products	74.11%	94.16%	69.54%	96.39%
					
ChEMBL Data	Valid JSON	98.80%	99.00%	99.20%	99.00%
	Template Mem.	99.90%	99.82%	99.37%	99.82%
	BB Selection	99.57%	99.23%	99.50%	99.47%
	Valid SMILES	92.02%	96.38%	95.86%	95.23%
	Matched Reactants	54.52%	69.25%	64.62%	70.93%
	Good Products	67.69%	85.03%	75.81%	87.02%

a We first select 1000 SMILES strings from the training examples, testing examples, and the ChEMBL dataset and then run inferences using SynLlama models trained on RXN 1. Benchmarking results for SynLlama models trained on RXN 2 can be found in Supplementary Table S1. The detailed descriptions of each benchmark can be found in the main text. Here, we run SynLlama inferences at *T* = 0.1 and *TopP* = 0.1 to generate reproducible benchmarking results (see Supplementary Table S2).

To assess SynLlama’s comprehension of reaction chemistry, we focus on individual reactions and summarize the three critical aspects as (1) the percentage of “Valid SMILES” out of all SMILES strings in the responses, which is essential for assessing SynLlama’s learning outcome of string-based chemical representations in general, (2) the percentage of “Matched Reactants”, which calculates whether the generated reactants match the reactant templates specified in the predicted reactions, and (3) the percentage of “Good Products”, which assess if the predicted product can indeed be generated by applying the proposed reaction templates onto the reactants. Overall, these six benchmarks can collectively assess SynLlama’s capability to follow instructions and perform chemical reactions in string representations.

In Table 1, all four trained SynLlama models are evaluated on both in-distribution training data and out-of-distribution testing and ChEMBL^8^ data to assess the benchmarks outlined above. In the instruction-following benchmarks, most models exhibit strong adherence (over 90%) to the fine-tuned response structure across all data sets. This impressive performance indicates that fine-tuning effectively retains the specified output structure when trained with over 100,000 samples. Furthermore, all four models successfully memorized the provided RXN templates and selected the building blocks (BBs) from all predicted reactants over 99% of the time. This capability further enhances the coupling effectiveness of the downstream reconstruction algorithm with the SynLlama raw output, as it requires information only about reaction sequences and predicted building blocks.

In the reaction chemistry benchmarking results, a clearer trend emerges: models, regardless of their parameter size, show improved comprehension of reaction chemistry in all three data sets as the amount of training data increases. Notably, most models maintain their performance from training to testing data but exhibit a greater decline in “Matched Reactants” and “Good Products” performance when generalizing to the ChEMBL data. The reason behind this is that the testing data are generated in the same manner as the training data but with a different set of building blocks, while the ChEMBL data occupies a different chemical space, as previously noted by Gao et al.^54^ Despite the reductions in their performance for ChEMBL molecules, as shown in Supplementary Figure S2, SynLlama-8B-500k and SynLlama-1B-2M can still generate complete and valid syntheses over 50% of the time without any downstream processing. These results indicate that SynLlamas raw results alone have potential utility for synthesis planning for unseen drug-like molecules.

When comparing SynLlama-8B-500k and SynLlama-1B-500k, we observe that the larger model demonstrates better performance when it is trained on the same amount of data. Although additional training data could further improve the 8B model based on the current trend, its higher computational cost makes this pursuit less practical. However, as the fine-tuning computational costs for SynLlama-8B-500k and SynLlama-1B-2M require approximately the same A40-GPU hours, and given the comparable benchmark performance between them, we decided to move forward with SynLlama-1B-2M, simplified as SynLlama, for the subsequent tasks due to its faster inference speed.

### 2.4 Reconstruction from Predicted Retrosynthesis

Using the predicted sequence of RXNs and BBs from SynLlama responses, we can synthesize the proposed target molecule or close analogs by applying the predicted reaction templates to the BBs in the inferred order, as shown in black ink in Figure 1(d). In some cases, the predicted BBs match known Enamine BBs, ensuring that the resulting molecules remain within an established chemical space for synthesis. However, due to SynLlama’s generative nature, some predicted BBs are novel while still providing valid synthesis pathways. To ensure practicality, we only report new BBs that can be purchased from other suppliers identified by Molport.^69^ Therefore, while SynLlama primarily produces molecules within the predefined chemical space using Enamine BBs, its output also offers an alternative strategy for molecule construction. We will revisit this point in the Results section.

When the input molecule cannot be fully reconstructed, we generate analogs by mapping the predicted BBs from SynLlama to known Enamine BBs, thereby sampling molecules from the well-defined Enamine chemical space. Under this scenario, we use nearest neighbor search algorithms with different molecular representations (SMILES and Morgan Fingerprints^70^) to sample Enamine neighboring BBs from the predicted BBs, as illustrated in colored inks in Figure 1(d). Since in SynLlama’s output, the RXN sequences are predicted concurrently with the BBs, our effective search space is constrained to Enamine BBs that can react through the specific RXN template. This smaller Enamine search space not only allows us to ensure the success rate of such forward syntheses but also allows us to effectively explore segments of the input molecule. Further details of the nearest neighbor search algorithms are provided in the Supporting Information.

When constructing full synthetic pathways for reactions with multiple possible products, we select the product that most closely matches the predicted product based on SMILES string similarity. As shown in Figure 1(e), the reconstruction algorithm iteratively builds synthesis routes using all predicted BBs and RXN sequences to reconstruct or generate variations of the original molecule from the synthesizable chemical space. This reconstruction algorithm enables the SynLlama model to function as a generator for synthesizable molecules along with their corresponding synthetic pathways.

## 3 Results

We examine SynLlama’s performance in synthesis planning of a diverse set of previously unseen compounds. We also explore the utility of the SynLlama workflow in real-world drug discovery applications, including its integration with generative algorithms to enhance the synthetic accessibility of proposed molecules while preserving their chemical properties and expanding the library of active compounds in the defined synthesizable space with good or improved binding affinity metrics.

### 3.1 Synthesis Planning for Unseen Molecules

Having demonstrated that SynLlama models can reliably predict reaction sequences and building blocks in Table 1, we now use SynLlama to plan the synthesis of two groups of 1000 previously unseen molecules from the Enamine Diversity Set^9^ and the publicly available ChEMBL database.^8^ These data sets are specific to drug-like molecules, unlike the training data used for SynLlama; the drug-related property distribution of both sets of molecules against the training data is shown in Figure S3. In this validation, we test whether known synthesizable molecules can be reconstructed accurately from the baseline and SynLlama models, as summarized in .

We first consider the standard reconstruction approach used by algorithms such as SynNet,^54^ ChemProjector,^55^ and Synformer^56^ to create target molecules or analogs using BBs exclusively from the Enamine library. In this comparison, SynNet^54^ and ChemProjector^55^ serve as a baseline comparison for synthesizable chemical space coverage for RXN 1, whereas Synformer^56^ is the baseline comparison on the expanded RXN 2 templates. As seen in the first column of Table 2 and Supplementary Table S3, when trained with their respective reaction sets and using only Enamine BBs, SynLlama outperforms all three methods while reducing the number of training data by 40- to 60-fold.

**Table 2:** Comparison of Synthesis Planning Performance among Different Methods^a^

		# of Recon. Mol.			
Data Set	Method	Enamine BB	New BB	Total	Morgan Sim.
Enamine Diversity Set	SynNet	110	-	110	0.57
	ChemProjector	462	-	462	0.82
	Synformer	660	-	660	0.91
				
	SynLlama (RXN 1)	527	100	568	0.87
	SynLlama (RXN 2)	691	232	741	0.92
					
ChEMBL Data	SynNet	54	-	54	0.43
	ChemProjector	133	-	133	0.60
	Synformer	198	-	198	0.67
				
	SynLlama (RXN 1)	165	95	223	0.66
	SynLlama (RXN 2)	197	152	287	0.68

a Both the Enamine Diversity Set and ChEMBL Data are comprised of 1000 unseen molecules each. Details of each benchmark are described in the main text. The Morgan similarity scores include all analog molecules with successful synthesis pathways, as well as successfully reconstructed target molecules. The number of total training data and reaction set each method used for is SynNet^54^ (200K, RXN 1), ChemProjector^55^ (128M, RXN 1), Synformer^56^ (85M, RXN 2), and SynLlama (2M, RXN 1 and 2). Further details about the properties of Synllama-predicted building blocks are provided in Supplementary Tables S4 and S5, and the procedure to identify purchasable New BBs with Molport^69^ is described in the Supporting Information.

Although SynLlama yields higher percentages of successful ChEMBL reconstructions compared to SynNet and ChemProjector and is on par with Synformer when only using Enamine BBs, there is a degradation of performance across all methods for ChEMBL compared to the Enamine Diversity set reconstructions. We attempted to improve upon the ChEMBL result by reformulating the training reaction data with extra filtering such that the product molecule distributions conform to a similar drug-like property distribution as the ChEMBL set, as seen in Supplementary Figure S4. We then performed supervised fine-tuning following the same procedure as described in Section 2, but now with this filtered set of training data, in the hope of better generating synthetic pathways for molecules with drug-like properties. However, as shown in Supplementary Table S3, there is now a performance loss over both the Enamine Diversity and ChEMBL drug-like targets. This result suggests that training on more diverse product molecules ultimately benefits synthesis planning for the drug-like targets more than specializing the LLM further with more restrictive training data. In addition, the curated ChEMBL data appear unique and outside the synthesizable chemical space made up of Enamine BBs and RXN templates.

However, unlike baseline methods that only generate molecules using Enamine BBs, SynLlama has the extra capacity to reconstruct target molecules with commercially available BBs beyond Enamine due to its generative capabilities, even without specific training for this purpose. As seen in Table 2 the “New BBs”, restricted to those purchasable through Molport,^69^ add possible synthetic pathways to reconstruct target molecules in all data sets and RXN templates. This also helps the ChEMBL data as well given that the molecules generated with the new BBs remain drug-like (Supplementary Figure S5). Since some target molecules can be synthesized through multiple pathways, either using only Enamine BBs or with the addition of New BBs, the “Total” column in Table 2 reflects the number of unique target molecules reconstructed with SynLlama. With these New BBs, SynLlama’s best reconstruction rates increase to 74.1% for the 1000 molecules in the Enamine Diversity Set, and encouragingly to 28.7% for the ChEMBL data. These results show that SynLlama learns reaction chemistry such that it can predict novel BBs to increase the synthetic accessibility.

When the target molecule cannot be reconstructed, we assess the quality of the analog using a molecular similarity score between the target molecule and its most similar analog using Tanimoto similarity based on 4096-bit Morgan fingerprints.^70^ In Table 2, the similarity metrics reported are average values of all generated molecules, including target molecules that are fully reconstructed (with a score of 1). Tables S6 and S7 also provide similarity metrics based on the 4096-bit Morgan fingerprints of Murcko scaffolds^71^ and Gobbi 2D pharmacophore fingerprints,^72^ while including or excluding target molecules that are fully reconstructed. Overall, these results collectively show that SynLlama is highly capable of planning synthesis for related analog molecules with very good similarity, aided most by increased synthetic pathways using purchasable building blocks.

Finally, Supplementary Table S3 also considers reconstruction performances for the Enamine Diversity Set and ChEMBL Data based on forward synthesis as opposed to retrosynthesis and for test molecules derived from tree-like synthesis pathways. It is evident that SynLlama performs better for retrosynthesis relative to forward synthesis that is used more successfully by Synformer, and retrosynthesis also performs better than the baseline methods for branching synthetic pathways.

### 3.2 Synthesizable Analog Search for *De Novo* Molecules

Previous research has shown that molecules proposed by generative models for binding to protein targets often face challenges in both reliability and practical synthesizability.^28–30^ In particular, medicinal chemists are often reluctant to devote time and expensive resources to the specialized synthesis of hit molecules due to the high false positive rates arising from the unreliability of docking scores in drug discovery. Instead finding closely related compounds that are constrained to Enamine BBs allows for an inexpensive purchase to verify hits before further refinements are deployed to gain lead drug molecules. In this section, we demonstrate SynLlama’s potential to bridge the gap between generative molecule design and practical synthesis for molecules that are optimized for drug-like applications such as binding assays.

Specifically, we consider two different generative methods for *de novo* drug molecules in Figure 2: a 1D-to-3D LSTM model, iMiner,^19^ which optimizes drug-likeness and AutoDock-vina^73^ docking scores, and Pocket2Mol,^22^ a 3D graph transformer model which also optimizes AutoDock-vina^73^ docking scores. Using iMiner we generated 500 molecules for the SARS-CoV-2 Main Protease (SARS2 MPro)^74^ and used Pocket2Mol to generate 500 molecules each for the protein targets Thrombin^75^ and TYK2.^76,77^ The first interesting observation is that only 1% of generated molecules from both iMiner and Pocket2Mol can be synthetically reconstructed using SynLlama or ChemProjector, suggesting that the generative models create difficult synthesis targets. Hence all 500 compounds for each protein target were then processed through SynLlama trained on RXN 2 to generate synthesizable analogs constrained to Enamine BBs. For all generated analogs, we performed molecular docking with AutoDock-vina via using the same protocol as the original binders.

**Figure 2:**
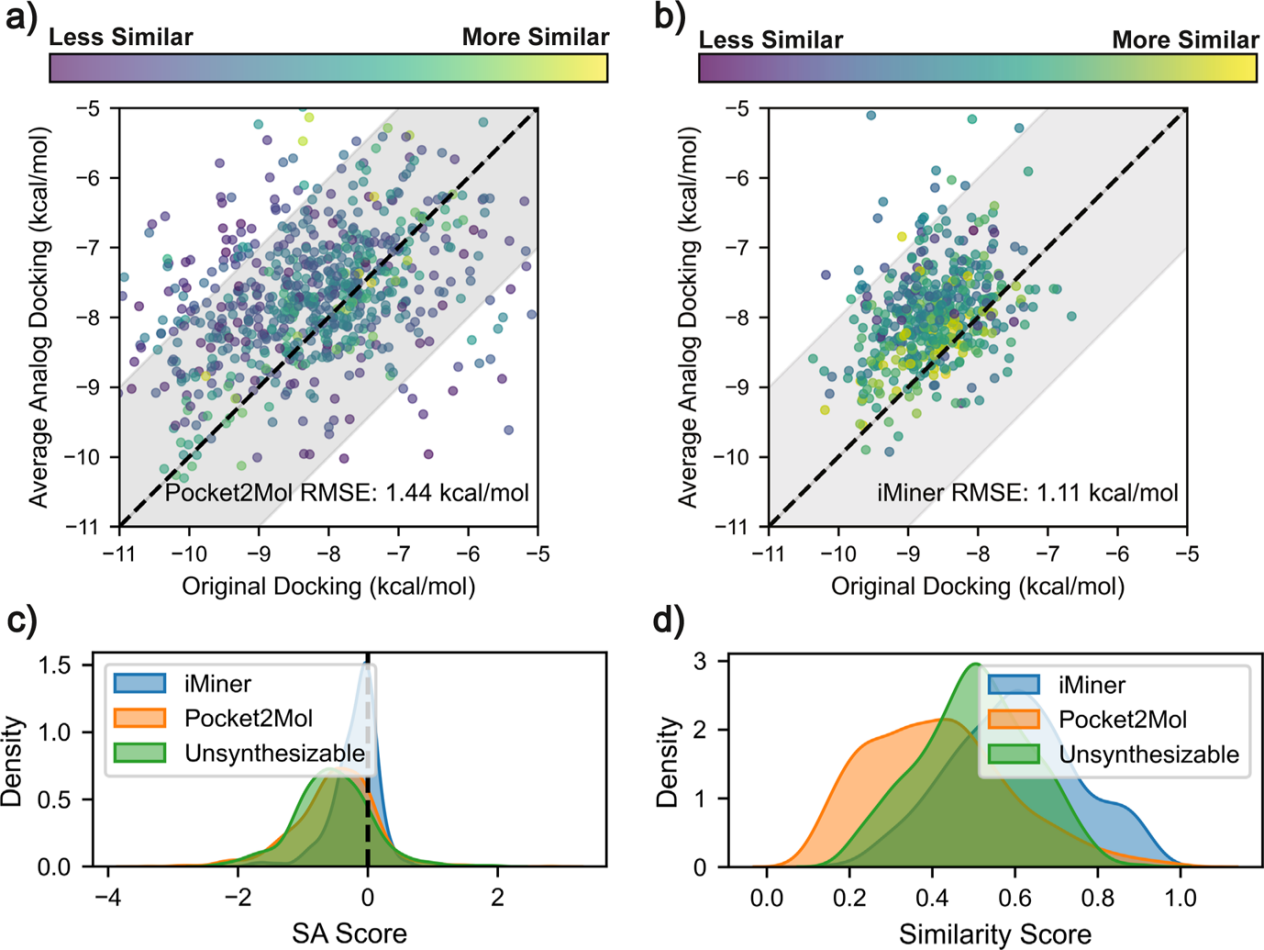
**SynLlama performance on generating synthesizable analogs for Pocket2Mol and iMiner proposed binders of SARS2 MPro**,^74^ **Thrombin**,^75^ **and TYK2**.^76,77^ Correlation plot comparing docking scores of (a) Pocket2Mol and (b) iMiner generated molecules and the average Vina docking scores of the ten most similar analogs from SynLlama trained with RXN 2. Each data point is color-coded by the average Morgan fingerprint similarity computed between the generated and analog molecules. The shaded area represents an energy uncertainty range of ±2 kcal/mol for docking.^73^ (c) The distribution of difference in synthetic accessibility (SA) score between Pocket2Mol, iMiner, and unsynthesizable molecules and SynLlama-proposed analogs. iMiner analogs generated with SynLlama trained on RXN 1 showed similar results as reported in Supplementary Figure S6. The kernel density in Supplementary Figure S7 further confirms our finding that the analogs consistently shift toward better SA without undermining the overall docking score distribution. (d) Average Morgan fingerprint similarity score between the target molecules and their top-10 proposed analogs.

Figure 2(a,b) shows the RMSE of the docking scores between the target compounds and the generated analogs are 1.44 and 1.11 kcal/mol for Pocket2Mol and iMiner, respectively, both of which are within the acceptable range of inherent docking score errors reported by Trott et al.^73^ Furthermore, as demonstrated in Figure 2(c,d), the SA score distribution of the SynLlama analogs generated for thrombin and TYK2 from Pocket2Mol shows a notable improvement in synthetic accessibility at the expense of reduced similarity below the benchmarks in Table 2. SynLlama slightly improves SA for iMiner generated compounds for SARS2 MPro while maintaining a good similarity score on par with the benchmarks in Table 2. The fact that iMiner uses a drug-likeness score as part of its loss function^19^ may explain its better performance, or perhaps SARS2 MPro is an easier protein target than Pocket2Mol’s thrombin and TYK2 protein targets.

The better synthesizable analogs have good retention of the binding mode of the original generated molecules, visually confirmed by the representative docking poses for target–analog molecular pairs shown in Figure 3(a) for the three proteins. Figure 3(b,c) provides a few examples of the synthesis pathways for the analogs of the molecules derived from the generative molecules.

**Figure 3:**
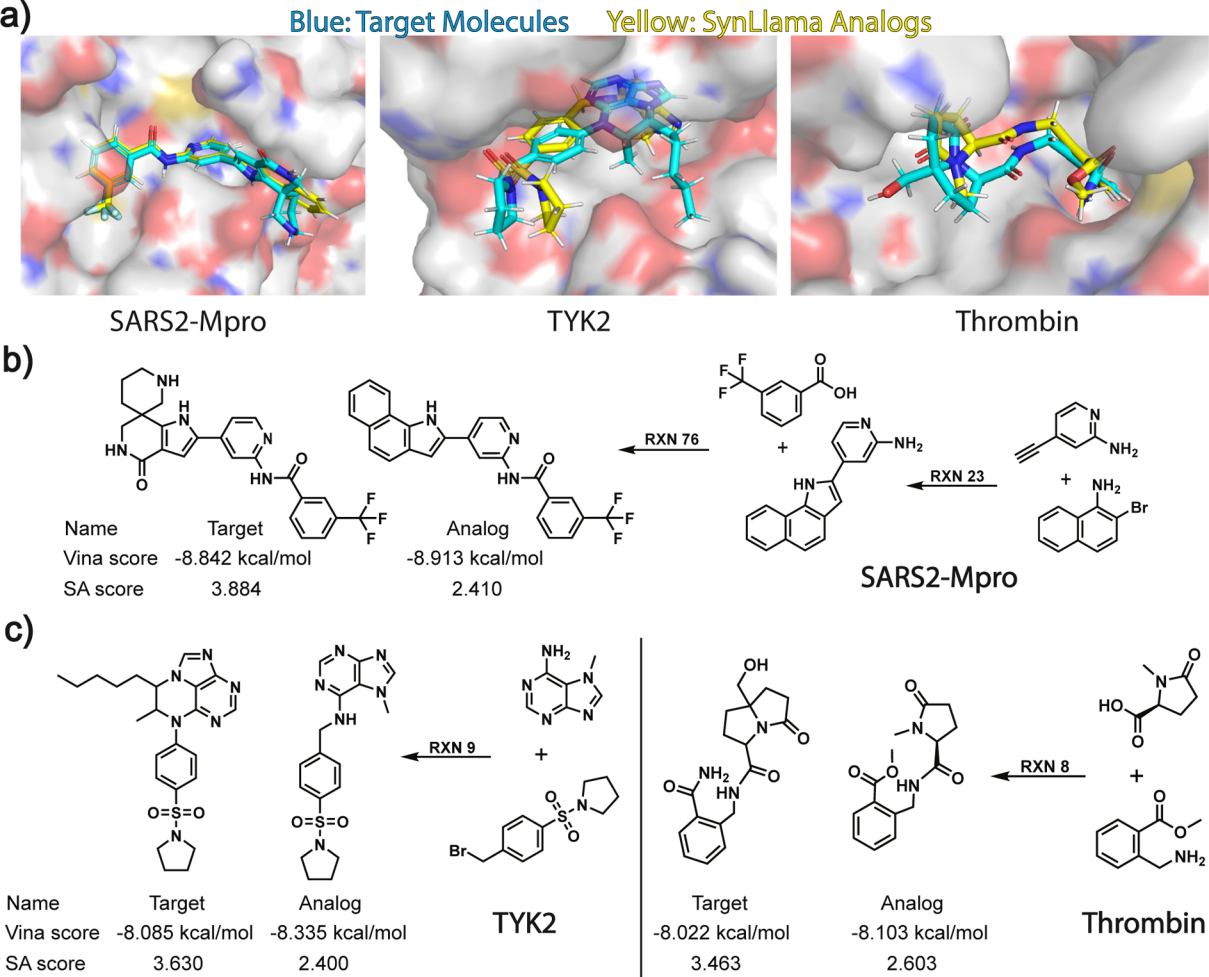
**Examples of synthesizable analog generation for SARS2 MPro using iMiner and TYK2 and Thrombin with Pocket2Mol**. (a) Docked pose visualization for all three protein targets. (b) Docking and SA scores for iMiner target and SynLlama analog for SARS2 MPro along with the predicted synthetic pathway. (c) Docking and SA scores for the Pocket2Mol targets and the SynLlama analogs for TYK2 and thrombin along with their predicted synthetic pathways.

The final comparison consists of molecules that were identified as unsynthesizable by Gao et al.^30^ via ASKCOS.^48^ As also seen in Figure 2, the similarity scores of SynLlama analogs for this set are better than that observed for the Pocket2Mol generative model, and the generated analogs show a significant decrease in SA score, representing SynLlamas effective strategy of improving synthetic accessibility via analog generation. In Supplementary Figure S8, we also highlight the few concrete examples of target–analog pairs where objective scores are maintained while target synthetic accessibility scores are substantially reduced. Overall, these collective results highlight SynLlama’s utility in effectively proposing synthesizable analogs for *de novo* molecules, thereby enhancing their synthetic accessibility without compromising their desired drug-related properties.

### 3.3 Local Hit Expansion for Binder Molecules

Because SynLlama breaks down the original target molecule for synthesis into building blocks, by nature, this method allows diverse exploration around parts of the molecular scaffold rather than only on a whole target molecule. In a final task, we apply SynLlama to expand on hit molecules for three protein targets used in the previous section, SARS2 Mpro,^78^ Thrombin,^75^ and TYK2 ^76,77^ to discover synthesizable molecules that have better relative binding free energies (RBFEs) confirmed by both experiments and accurate free energy perturbation (FEP) calculations.

As shown in Figure 4(a), the hit molecule for SARS2 MPro (7LTJ_Lead) has a core scaffold of uracil and orthodichlorobenzene connected by a piperizine linker. Inspired by an experimental hit expansion campaign by Kneller et al.,^79^ we follow their practice to propose only functional group substitutions on the benzene ring while keeping the linker and uracil intact. In Figure 4(c,e), we use the best-performing molecules from the Schrodinger FEP benchmarking set^80^ as our hits for Thrombin and TYK2. To expand on these hit molecules, we first identify the maximum common scaffolds among each group of molecules in the FEP benchmarking set and use the identified scaffolds to guide our selection of analog molecules. Specifically, we use SynLlama to generate 50 synthesizable analogs constrained to only Enamine BBs of the hit compound and filter for molecules that retain the scaffold. In the end, we harvest a total of 8, 14, and 11 analog molecules that fulfill the criteria for SARS2 MPro, Thrombin, and TYK2, respectively. The analogs are then placed in a pose configuration similar to the original hit molecule for downstream FEP calculations.

**Figure 4:**
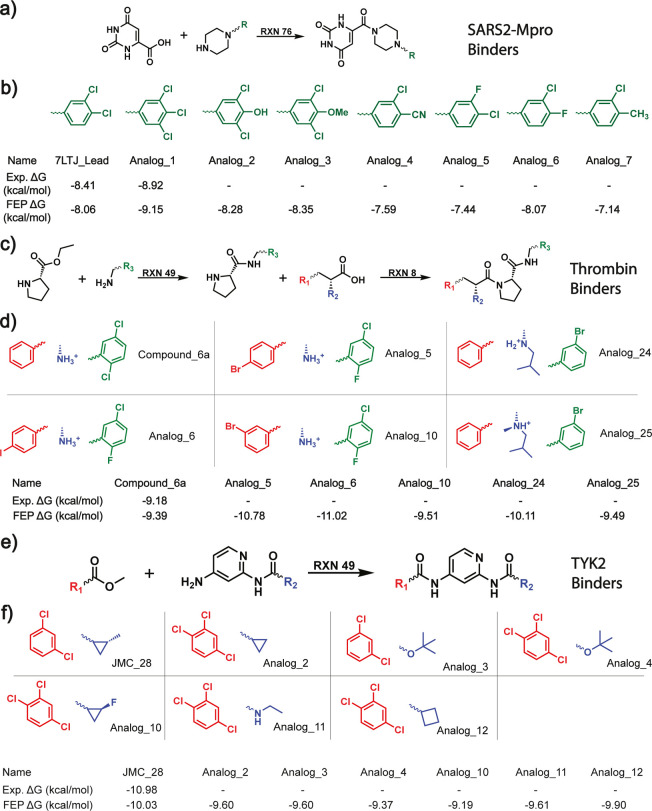
**Hit expansion of binders to SARS2 Mpro, Thrombin, and Tyk2 with SynLlama.** (a,c,e) Synllama-predicted synthetic pathways that expand on the hit molecules for each protein target. The places of substitution are labeled as R groups. (b,d,f) Binding free energies of the hit compounds and SynLlama-expanded analogs. The color scheme of the proposed substitution is the same as the predicted synthetic pathways. All potential binders either have a better FEP binding free energy or are within the 1 kcal/mol uncertainty range compared to the original hits.

To verify the FEP results, we first choose ∼10 synthesized and experimentally tested molecules to benchmark the accuracy of the FEP for all three systems. In Supplementary Figure S9, all the calculated FEP values show a good correlation with the experimental *ΔG* converted from IC50, with an average RMSE of less than 1 kcal/mol. After this validation, we run FEP for all of the proposed molecules to assess their binding affinities. As shown in Figure 4(b,d,f), a significant portion of the proposed analogs (7 of 8, 5 of 14, and 6 of 11, respectively) showed potency compared to their parent hits for SARS2 MPro, Thrombin, and TYK2. These results successfully demonstrate that SynLlama can propose diverse yet potent analogs when constrained on a molecular scaffold.

Moreover, the fact that all suggested analogs also come with predicted pathways using common reaction templates and purchasable BBs from Enamine suggests SynLlama’s practical use for drug discovery. Because of this composite capacity of optimizing both potency and synthetic accessibility for small molecules, SynLlama successfully rediscovered Analog_1, the most potent compound reported by Kneller et al. in their hit expansion campaign for SARS2 MPro.^79^ Furthermore, for the Thrombin case, SynLlama explores the R2 substitution site, a region previously unaddressed by earlier molecular series and demonstrates the generation of more potent molecules via FEP. These results confirm that SynLlama effectively explores local chemistry with readily available building blocks, providing a direct and efficient path for medicinal chemists to accelerate hit expansion.

## 4 Discussion and Conclusions

Motivated by recent advances in LLMs for chemistry,^27,58–60^ we aim to leverage data-efficient supervised fine-tuning (SFT) to transform the general-purpose Meta Llama 3 into SynLlama, an LLM-based generator capable of proposing synthesizable molecules and deducing synthetic routes for target molecules or their close analogs. Throughout the study, we successfully show that SynLlama can effectively explore a custom-defined chemical search space composed of around 230,000 Enamine building blocks (BBs) and well-validated organic reactions (RXNs), after it has been fine-tuned on synthetic pathway data sampled from this specified chemical space. What’s more, despite utilizing nearly 2 orders of magnitude fewer synthetic pathways in training, SynLlama exhibits strong performance in key drug discovery tasks compared to existing models. Specifically, we have demonstrated that SynLlama can effectively aid in various stages of drug discovery that include synthesis planning, synthesizable analog generation for *de novo* molecules, and local hit expansion.

Because SynLlama is built on a general-purpose LLM instead of training from scratch,^54–56^ it offers a number of unique advantages and possibilities for further improvement. For example, when generating our fine-tuning data, we sampled from the predefined chemical search space of 230 K Enamine building blocks (BBs) and two sets of reaction templates (RXNs), but we did not embed these extensive requirements in the context window of the LLM. As a result, for our largest data set with only 2 million synthetic pathways, the model only saw each BB a few dozen times, while each RXN template appeared hundreds of thousands of times. Consequently, while SynLlama efficiently memorizes the allowed RXNs, it only captures the distribution of Enamine BBs, which enables SynLlama to extrapolate to unseen yet purchasable building blocks outside of Enamine. This generative ability surpasses other existing methods such as ChemProjector and Synformer that can only explore a predefined building block search space, such as within the Enamine Diversity Set,^9^ and limits their ability to propose alternative synthesis pathway with novel building blocks.

In addition to its ability to extrapolate outside the training chemical space, the underlying Llama-3.2-1B used by SynLlama is relatively small, and more predictive power would be expected if we train on larger LLMs with more data and compute power. However, we observe that a smaller LLM with fewer parameters can be turned into an expert model for complex tasks after SFT with relatively little data. This opens up opportunities to employ smaller expert models for various chemical tasks, benefiting from faster inference speeds, which can make these models more desirable. Moreover, optimal hyperparameters like temperature and top-p can vary between training and inference phases depending on the downstream tasks. During inference, most valid raw outputs are generated under relatively low temperatures and top-p settings. However, when the model is paired with reconstruction algorithms that require less strict adherence to the reaction chemistry, higher temperature and top-p values can be used. This allows for a broader exploration of the Enamine chemical space, enabling the generation of more diverse and relevant analogs. This property is especially desirable in tasks that require extensive exploration, such as the hit expansion example that we demonstrate. Another exciting direction is coupling SynLlama with another generative model, in which we have shown generates analogs while maintaining good docking scores and simultaneously shifting to better synthetic accessibility scores. This result suggests that SynLlama can serve effectively as a postprocessor for other *de novo* generative models, ensuring the production of more synthesizable compounds with clear reaction pathways.

Among the numerous opportunities that LLMs bring to the field of drug discovery, their natural language capabilities and recent advancements in reasoning are exciting features that allow users without coding expertise to interact directly with the models, effectively bridging the gap between computational methods and experimental research. We envision that expert users can employ prompt engineering and fine-tuning of data to incorporate more realistic factors than those explored here. For instance, medicinal chemists could fine-tune LLMs within this generalizable SFT framework with building blocks and reaction templates of their own choice. In addition, they can consider the synthesis cost, reaction conditions, improved selectivity, and protection factors at specific reaction steps for more detailed and powerful synthesis planning. We see our work as an initial attempt to demonstrate the effectiveness of LLMs in real experimental research, encouraging further studies for better utilization of these models.

## Supplementary Material



## Data Availability

All the codes and data for the SynLlama workflow are provided in a public accessible GitHub repository, https://github.com/THGLab/SynLlama, under MIT license.

## Notes

Additional methodology and results, including details of SFT protocols; generation of training reaction data and hyperparameters used during inferences; baseline benchmarking procedures and results; procedures to check the purchasability and overall drug-related property distribution of novel building blocks; procedures to generate *de novo* molecules via iMiner and Pocket2Mol; details to calculate various rewards functions (docking scores, SA scores, and FEP energies); results of additional benchmarking on reconstruction, LLM reliability, and analog similarities; and the effect of different hyperparameters on LLM inferences and downstream Enamine reconstructions (PDF)
