# Supplementary material for: SynLlama: Generating Synthesizable Molecules and Their Analogs with Large Language Models
Source: ACS Cent Sci. 2025 Sep 17;11(11):2108–20. doi: 10.1021/acscentsci.5c01285 (PMC12670306; doi:10.1021/acscentsci.5c01285)
Supplement: Supplementary file 1 [file oc5c01285_si_001.pdf]

Supplementary Information SynLlama: Generating Synthesizable Molecules and Their Analogs with Large Language Models

Kunyang Sun[1], Dorian Bagni[1,Δ], Joseph M. Cavanagh[1,Δ], Yingze Wang[1,Δ], Jacob M. Sawyer[4], Bo Zhou[5], Andrew Gritsevskiy[6], Oufan Zhang[1], Teresa Head-Gordon*[1−3]

[1]Kenneth S. Pitzer Theory Center and Department of Chemistry, [2]Department of Bioengineering, [3]Department of Chemical and Biomolecular Engineering, University of California, Berkeley, CA, 94720 USA
[4]Department of Chemistry, University of Minnesota, 207 Pleasant Street SE, Minneapolis, MN 55455, USA [5]Contramont Research, San Francisco, CA, 94158 USA
[5]Department of Pharmaceutical Sciences, University of Illinois Chicago, 833 S Wood St, Chicago, IL 60612, USA
[6]Contramont Research, San Francisco, CA, 94158 USA
[Δ]authors contributed equally
corresponding author: thg@berkeley.edu

Additional Methodology Details

Supervised Fine Tuning protocol. After preparing the reaction data and prompt-response pairs from the training chemical space, we fine-tune Llama-3.1-8B (8 Billion parameters) and Llama-3.2-1B (1 Billion parameters) using the Axolotl package[1,2] for 1 epoch. LLMs with more parameters require more resources to train and use, but they also typically perform better on a variety of tasks, which we consider in Results. For our SFT approach, we apply Low-Rank Adaptation (LoRA) with a rank of $r = 32$ and $\alpha = 16$ to the linear layers of the model.[3] We use FlashAttention-2[4], with the Adam optimizer[5], cross-entropy loss, and a cosine learning rate scheduler with a maximum learning rate of 2×10^{-5}.

Molecule Generation using Enamine BBs. When searching for the nearest neighbors of BBs, a natural choice is to perform a string-level similarity search based on SMILES strings, as this is the native format of SynLlama responses. For each RXN template, we systematically process all SMILES strings of its compatible building blocks that can participate in the reaction. First, we extract the full vocabulary of SMILES tokens and generate an n-gram representation by considering all possible consecutive token pairs (bigrams) and triplets (trigrams). Next, we identify the 1024 most frequently occurring n-grams across

SMILES strings of all compatible BBs to form a representative token set for each individual RXN template. To facilitate efficient retrieval, we structure search trees based on the term frequency-inverse document frequency (TF-IDF) scores[6] of these n-grams, prioritizing highly informative substructures and accelerating inference. Consequently, when a new SMILES string of the predicted BB is introduced, it can be efficiently processed through the tree, yielding a list of the top K matching SMILES strings.

In addition, Gao et al.[7] investigated using Morgan fingerprints[8], a molecular representation capturing local chemical environment, to search for the nearest neighbors of BBs based on their Tanimoto similarity[9]. Similarly to that stated above, for each RXN template, we also build a separate search tree for all compatible Enamine BBs using 256-bit Morgan fingerprint representation with a searching radius of 2. Our empirical observations indicate that combining the top K molecules from both the SMILES and Morgan fingerprint methods offers better performance than relying on the top 2K molecules from a single method. However, since we are working with an LLM model, the generated SMILES strings still have a small chance of being invalid, which prevents us from calculating their Morgan fingerprints. Therefore, we employ the both combined TF-IDF and Morgan fingerprint search trees when dealing with valid molecules, and revert to only a SMILES-based search when the generated SMILES strings are invalid.

LLM Inference Hyperparameters for Various Tasks. A key advantage of SynLlama, and LLMs in general, is their sensitivity to variations in hyperparameters, such as temperature (T) and top-p ($TopP$), which can significantly impact the performance of reconstruction and analog similarity. As shown in Supplementary Figure S2, SynLlama's raw outputs exhibit enhanced reaction chemistry comprehension when inferences are run at lower $TopP$ and within a reasonable range of T for both test sets. This configuration allows SynLlama to explore purchasable building blocks outside the Enamine library while maintaining synthesis validity. Conversely, increasing T and $TopP$ generally reduces SynLlama's ability to generate valid syntheses in its raw outputs. However, as Supplementary Figure S2 also illustrates, inferring with higher T and $TopP$ values than the optimal settings in raw outputs often leads to better overall average maximum similarity scores for reconstruction with Enamine BBs along. Nonetheless, excessively high settings can increase the failure rate.

Based on empirical observations, we recommend specific combinations of T and $TopP$ that effectively span a broad spectrum of tasks. These combinations optimize SynLlama's performance by balancing exploration and precision during inference.

- **Frozen**: $T = 0.1, TopP = 0.1$, repeated once. This setting prioritizes deterministic generation, ensuring minimal variability and high reproducibility.

- **Low**: $T = 0.6, TopP = 0.5$, repeated multiple times. This configuration allows for limited exploration while maintaining a degree of precision.

- **Medium**: $T = 1.0, TopP = 0.7$, repeated multiple times. This setting balances exploration and diversity, generating outputs with moderate randomness.

- **High**: $T = 1.5, TopP = 0.9$, repeated multiple times. This configuration promotes high diversity and creativity in generation but may introduce more variability in results.

We define different sampling strategies based on these core settings:

- **Frugal Sampling**: A total of 4 inferences.
  - $T = 0.1, TopP = 0.1$, repeated one time.
  - $T = 0.6, TopP = 0.5$, repeated one time.
  - $T = 1.0, TopP = 0.7$, repeated one time.
  - $T = 1.5, TopP = 0.9$, repeated one time.
- **Greedy Sampling**: A total of 10 inferences.
  - $T = 0.1, TopP = 0.1$, repeated one time.
  - $T = 0.6, TopP = 0.5$, repeated two times.
  - $T = 1.0, TopP = 0.7$, repeated three times.
  - $T = 1.5, TopP = 0.9$, repeated four times.
- **Frozen Only**: A total of 1 inference.
  - $T = 0.1, TopP = 0.1$, repeated one time.
- **Low Only**: A total of 5 inferences.
  - $T = 0.6, TopP = 0.5$, repeated five times.
- **Medium Only**: A total of 5 inferences.
  - $T = 1.0, TopP = 0.7$, repeated five times.
- **High Only**: A total of 5 inferences.
  - $T = 1.5, TopP = 0.9$, repeated five times.

Baseline Benchmarking Details. Since the Enamine BB catalog constantly updates new BBs and does not store historical data, we cannot access the exact training BBs used in training for the baseline methods ChemProjector[10] and Synformer[11]. The only training set data we had access to is described in Section 2.1 in the main document, and is $\sim 3\%$ (10k) more compared to that available to Synformer and Chemprojector (cutoff at October 2023). However, we highlight that 97% of our training data is identical to their previous work and the newly added building blocks (which is equivalent to a time split) show a similar distribution as the rest of the training BBs. We now have this comparison in Supplementary Figure S5. Therefore, we can fairly say that our training data are very similar to the baseline methods to which we compare. For a fair comparison at the inference stage, we provide ChemProjector and Synformer the same set of building blocks (cutoff at Feb. 2025) that SynLlama had access to during inference time. In Tables 2 and S3, we assess the performance of the trained baseline models with this more recent set of building blocks.

Checking Commercial Availability of Building Blocks via Molport. In Results, we used the Molport platform to check whether a predicted BB is commercially available or not. Initially, we compiled a list of building blocks for searching and used the 'List Search' tab in the Molport website (https://www.molport.com/shop/swl-step-1) to check their availability. Once the SMILES strings were entered into the search interface, we set the search criteria to a minimum acceptable quantity of 500 mg and match types restricted to 'Exact' and 'Perfect' to search in the database of 'screening compounds' and 'building blocks.' Once the search completed, we downloaded the excel file under the 'Selected Items' column from the List Search result tab (https://www.molport.com/shop/swl-requests), which contained both the commercially available compounds and information about the supplying vendors.

Calculation of SA Scores. We calculate SA scores for both the iMiner-proposed molecules and SynLlama-generated analogs using the oracle functions named 'SA' implemented in the TDC Commons package[12].

iMiner-Generated Molecules and Docking Procedures for Analogs. The iMiner algorithm[13], an 1D string-based LSTM model for SELFIES[14] string generation, was employed in this study. The molecules generated by iMiner are optimized for 3D shape complementarity using a composite objective function comprising the AutoDock Vina[15] docking score, as well as a custom-defined druglikeness score[13].

For molecular docking tasks, we obtained the SARS-CoV-2 Mpro crystal structure (PDB ID: 7L11[16]) from the Protein Data Bank[17] and processed it with PDBFixer[18] to add missing hydrogens and remove heteroatoms. The docking grid was centered at the geometric center of the ligand (XF1) from the corresponding PDB file ([x = -22, y = -4, z = -28]) using a cubic box with 20 Å sides. Both proteins and ligands were converted to PDBQT format using Meeko (https://github.com/forlilab/meeko). Docking was performed with AutoDock Vina using an exhaustiveness parameter of 64, and the best pose for each ligand was recorded. This protocol was consistently applied during both iMiner training and analog docking assessments.

The custom drug-likeness score is a composite score that evaluates 13 key molecular properties derived from the ChEMBL database. These properties capture both basic structural features and nuanced physicochemical characteristics, including the fraction of sp^3-hybridized carbons, the total number of heavy atoms, and the fraction of non-carbon atoms within these heavy atoms. Additionally, the score accounts for the counts of hydrogen bond donors and acceptors, the number of rotatable bonds, and the balance between aliphatic and aromatic rings, along with molecular weight. Complementing these are parameters such as the approximate log partition coefficient (alogP), polarizable surface area (PSA), the number of structural alerts, and the size of the largest ring present in the molecule. Each property contributes to the overall score through a weight that is inversely proportional to the entropy of its distribution in the ChEMBL database: properties with narrower and more informative distributions exert a stronger influence. By summing the log likelihoods of these properties with their respective weights, the score effectively biases the generative model to produce molecules that closely mimic the drug-like profiles observed in established therapeutics, ensuring that the exploration of chemical space remains focused on compounds with favorable bio-availability and efficacy profiles.

Pocket2Mol Generation. *De novo* generation with Pocket2Mol was performed for Thrombin and TYK2 targets using codes from the Pocket2Mol github repository[19]. Three default settings specified in `configs/sample_for_pdb.yml` were modified to generate at least 1000 molecules in one single run: `num_samples:1000, beam_size:500, max_steps:100`. The protein structure files were downloaded from the Schrödinger FEP benchmark github repository[20]. The pocket center was set to (-4.0, 26.5, -30.0) for TYK2 and (17.0, -12.5, 22.5) for Thrombin.

Unsynthesizable Molecules Identificaiton and Reward Calculation. To access the list of the unsynthesizable molecules, we query the first 50 top-scoring molecules that were identified as unsynthesizable by ASKCOS[21] for each property category listed in this csv (`https://github.com/wenhao-gao/askcos_synthesizability/blob/master/results/goal_hard_cwo.csv`). There are a total of 10 different individual rewards, including 7 multi-property objectives (MPOs) centering around 7 different drug targets (Osimertinib, Fexofenadine, Ranolazine, Perindopril, Amlodipine, Sitagliptin, Zaleplon), Valsartan SMARTS, and 2 Hopping (Scaffold and deco). We used the TDC Commons package[12] to score both the original molecules and the generated analogs for their corresponding property category.

Free Energy Perturbation (FEP) Protocols. The relative binding free energies are calculated using GPU-accelerated AMBER22[22] (`pmemd.cuda.MPI`). AMBER14SB[23] and OpenFF-2.1.0[24] were used to parametrize the protein and the ligand, respectively. The SARS-CoV-2 Mpro protein structure (PDB code: 7LTJ) was downloaded from RCSB PDB and prepared with PDBFixer[25] to assign side-chain protonation states at pH=7.4 and add hydrogens. H163 was manually set to be its variant HIE (hydrogen added on Nϵ) to ensure the correct hydrogen bonding with the ligand. For TYK2 and Thrombin, their protein structures were downloaded from the github repository of Schrödinger benchmark dataset[20]. A submodule `app.Modeller` in OpenMM[26] was used to immerse the protein-ligand complexes and unbound ligands in a cubic water box with 15Å buffer size and add ions ($\mathrm{Na}^+, \mathrm{Cl}^-$) to neutralize the system and maintain 0.15M ionic strength. For perturbations involving charge changes, the alchemical water method[27] was used to eliminate the artifacts in PME simulation of system with net-charges.

We used 16 unevenly distributed lambdas (0.0, 0.174, 0.226, 0.265, 0.330, 0.383, 0.432, 0.477, 0.522, 0.568, 0.617, 0.670, 0.735, 0.774, 0.826, 1.0) to transform the initial state to the final state in the free energy. This lambda settings was designed to maximize the phase space overlap between adjacent states with the second-order smooth-step function introduced. The transformations were performed with the modified SSC(2) softcore potentials ($m = n = 2, \alpha_{\mathrm{LJ}} = 0.5, \alpha_{\mathrm{Coul}} = 1$)[28]. Kartograf[29] algorithm was used to determine the common core region (SC) and soft core region (SC) atoms.

Each lambda state was subjected to the following simulation protocol to equilibrate the system: (1) energy minimization without any constriants; (2) heating from 0 to 100 K at constant volume and temperature (NVT) ensemble over 20 ps, followed by MD at constant pressure and temperature (NPT) ensemble at 100 K for 20 ps; (3) heating to 200 K at NVT ensemble over 20 ps followed by another 20 ps at NPT ensemble at 200 K; (4) heating to 298.15 K at NVT ensemble over 20 ps followed by another 20 ps at NPT ensemble at 298.15 K; (5) another pre-prodcution equilibrium run at NPT ensemble for 500 ps. During

the equilibration steps 2-4, restraints ($5\ \mathrm{kJ} \cdot \mathrm{mol}^{-1} \cdot \mathrm{\AA}^2$) were applied to heavy atoms on the solute. Finally, a 5-ns production run was performed for each lambda state with the ACES enhanced sampling method[30] and replica exchange was attempted every 0.5 ps. All the simulations employed 4 fs time step with the mass of solute hydrogens repartitioned to 3 amu[31]. MBAR algorithm implemented in `alchemlyb`[32] was used to estimate the free energy change between two states and yield $\Delta\Delta G$. Then, the maximum likelihood estimation (MLE) method[33] was used to calculate the absolute binding free energy (ΔG) of each ligand and the ΔG was shifted to make the average of calculated ΔG of the ligands equal to the average of their experimental ΔG:

$$\sum_i \Delta G^{(i)}_{\mathrm{pred}} = \sum_i \Delta G^{(i)}_{\mathrm{expt}} = \sum_i RT \ln \mathrm{IC_{50}}^{(i)}$$

All the system preparation and analysis were performed with an in-house package named `easybfe` that automates the whole workflow and manage the calculations with high-performance computing platforms, and it will be described in a future publication in details.

Supporting Tables

Dataset	Category	SynLlama(RXN 1)	SynLlama(RXN 2)
Training Data	Valid JSON	98.00%	98.20%
	Template Mem.	100.0%	100.0%
	BB Selection	99.96%	99.96%
	Valid SMILES	99.46%	99.70%
	Matched Reactants	97.64%	97.95%
	Good Products	98.58%	97.97%
Testing Data	Valid JSON	93.90%	94.60%
	Template Mem.	100.0%	100.0%
	BB Selection	99.66%	100.0%
	Valid SMILES	99.50%	99.46%
	Matched Reactants	96.90%	97.25%
	Good Products	96.39%	96.19%
ChEMBL Data	Valid JSON	99.00%	99.00%
	Template Mem.	99.82%	100.0%
	BB Selection	99.47%	99.81%
	Valid SMILES	95.23%	97.33%
	Matched Reactants	70.93%	84.02%
	Good Products	87.02%	87.65%

Table S1: Benchmarks of SynLlama inferences using SynLlama models trained with two sets of reaction templates. Here, both models are fine-tuned on Llama-3.2-1B model with 2M reaction data generate using the same set of training building blocks. We select 1000 molecules for each model: training and testing data are generated using their corresponding reaction templates; ChEMBL data is the same set of 1000 molecules as described in the main text. All SynLlama inferences are run at $T = 0.1$ and $TopP = 0.1$.

**Task**	**Sampling Method**	K	N_{Syn}
LLM Benchmark	Frozen Only	5	25
Synthesis Planning	Greedy Sampling	5	25
Synthesizable Analog	High Only	10	50
Hit Expansion	High Only	20	100

Table S2: Hyperparameters used for each task. Under each task name we include the sampling method used for SynLlama inferences as defined in Additional Methodology Details. K represents the number of most similar SMILES string to take during the reconstruction algorithm. N_{Syn} represents the maximum number of synthesis routes to be tracked for each single SynLlama inference during the reconstruction algorithm.

Dataset	Method	# of Recon. Mol.			Morgan Sim.
		Enamine BB	New BB	Total	
Enamine Diversity	SynLlama	691	232	741	0.92
	SynLlama - druglike	574	100	595	0.86
	SynLlama - forward	529	50	546	0.85
ChEMBL Data	SynLlama	197	152	287	0.68
	SynLlama - druglike	124	107	197	0.61
	SynLlama - forward	132	58	168	0.59
Branching synthesis	ChemProjector	302	-	302	0.79
	SynLlama (RXN 1)	415	118	465	0.87
	Synformer†	39	-	39	0.61
	SynLlama (RXN 2)	358	101	408	0.84

† The released Synformer[11] model weights were fine-tuned extensively on smaller drug-like compounds, which caused it to fall short on synthesis planning for more complex molecules.

Table S3: Reconstruction performances across various reaction data sets and prompt design choices. SynLlama-druglike refers to fine-tuning on target molecule that falls within the same distribution as ChEMBL. More detailed analysis of the target molecule properties can be found in Figure S1. SynLlama-forward refers to fine-tune on prompt-response pairs structured as forward synthesis rather than retrosynthesis. SynLlama-branching refers to reconstructions based on tree-like synthesis with testing molecules generated similarly to the training data using testing building blocks and corresponding two sets of reaction templates. We compare to ChemProjector[10] (RXN 1) and Synformer[11] (RXN 2).

Dataset	% of BB in Enamine	# of Raw Reconstructed Mol.		
		Enamine BBs	New BBs	Total
Testing	75.85%	506	125	563
Enamine	73.51%	510	100	557
ChEMBL	48.07%	161	95	221

Table S4: Comparison of Enamine BB presence and reconstruction with purchasable BBs across datasets at greedy temperature and top-p combo when using 91 RXN templates (RXN 1).

Dataset	% of BB in Enamine	# of Raw Reconstructed Mol.		
		Enamine BBs	New BBs	Total
Testing	76.61%	465	114	520
Enamine	68.34%	647	232	711
ChEMBL	48.04%	179	152	280

Table S5: Comparison of Enamine BB presence and reconstruction with purchasable BBs across datasets at greedy temperature and top-p combo when using 115 RXN templates (RXN 2).

Dataset	Method	Morgan	Scaffold	Gobbi
Enamine Diversity	SynNet	0.57	0.57	0.52
	ChemProjector	0.82	0.85	0.83
	Synformer	0.91	0.92	0.89
	SynLlama (RXN 1)	0.87	0.88	0.85
	SynLlama (RXN 2)	0.92	0.94	0.92
ChEMBL Data	SynNet	0.43	0.20	0.27
	ChemProjector	0.60	0.59	0.56
	Synformer	0.67	0.72	0.72
	SynLlama (RXN 1)	0.66	0.67	0.63
	SynLlama (RXN 2)	0.68	0.69	0.66

Table S6: Similarity metric comparisons over all successful reconstructions of target and analog molecules. Similarity metrics using Morgan, Scaffold, and Gobbi similarity scores. SynNet[7] and Chemprojector[10] are trained using RXN 1, and Synformer[11] is trained using RXN 2. Scores are computed over successful reconstructions of target and analog molecules from Table 2.

Dataset	Method	Similarity		
		Morgan	Scaffold	Gobbi
Enamine Data	SynNet	0.51	0.51	0.45
	ChemProjector	0.67	0.72	0.69
	Synformer	0.74	0.76	0.69
	SynLlama(RXN 1)	0.69	0.72	0.65
	SynLlama(RXN 2)	0.69	0.75	0.70
ChEMBL Data	SynNet	0.39	0.38	0.22
	ChemProjector	0.54	0.52	0.49
	Synformer	0.59	0.65	0.65
	SynLlama(RXN 1)	0.56	0.57	0.51
	SynLlama(RXN 2)	0.54	0.56	0.52

Table S7: Similarity metric comparisons over analog molecules when target molecules could not be constructed. SynNet[7] and Chemprojector[10] are trained using RXN 1, and Synformer[11] is trained using RXN 2. Scores are computed over molecules from Table 2 that could not be fully reconstructed.

Supporting Figures

**Instruction**	You are an expert synthetic organic chemist. Your task is to design a synthesis pathway for a given target molecule using common and reliable reaction templates and building blocks. Follow these instructions:\n\n1. **Input the SMILES String:** Read in the SMILES string of the target molecule and identify common reaction templates that can be applied.\n\n2. **Decompose the Target Molecule:** Use the identified reaction templates to decompose the target molecule into different intermediates.\n\n3. **Check for Building Blocks:** For each intermediate:\n - Identify if it is a building block. If it is, wrap it in <bb> and </bb> tags and save it for later use.\n - If it is not a building block, apply additional reaction templates to further decompose it into building blocks.\n\n4. **Document Reactions:** For each reaction documented in the output, wrap the reaction template in <rxn> and </rxn> tags.\n\n5. **Repeat the Process:** Continue this process until all intermediates are decomposed into building blocks, and document each step clearly in a structured JSON format.
**Input**	Provide a synthetic pathway for this SMILES string:<br>Cn1ncc(-c2ccc3c(c2)CC(C(=O)NCCOCc2ccccc2Cl)C3)n1
**Output**	"{'reactions':<br>[<br>{'reaction_number': 1,<br>'reaction_template':<br>'<rxn>[Cl,OH,O-:3][C$(C(=O)([CX4,c])),C$([CH](=O)):2]=[O:4].[N$([NH2,NH3+1]([CX4,c])),N$([NH]([CX4,c])([CX4,c])):6]>>[N+0:6]-[C:2]=[O:4]</rxn>',<br>'reactants': ['NCCOCc1ccccc1Cl',<br>'Cn1ncc(-c2ccc3c(c2)CC(C(=O)O)C3)n1'],<br>'product': 'Cn1ncc(-c2ccc3c(c2)CC(C(=O)NCCOCc2ccccc2Cl)C3)n1'},<br>{'reaction_number': 2,<br>'reaction_template':<br>'<rxn>[Br:1][c$(c(Br)),n$(n(Br)),o$(o(Br)),C$([CH](Br)(=C)):2].[C$(C(B)([CX4])([CX4])([CX4])),C$([CH](B)([CX4])([CX4])),C$([CH2](B)([CX4])),C$([CH2](B)),C$(C(B)(=C)),c$(c(B)),o$(o(B)),n$(n(B)):3][B$(B([C,c,n,o])([OH,$(OC)])([OH,$(OC)])),B$([B-1]([C,c,n,o])(N)([OH,$(OC)])([OH,$(OC)])):4]>>[C,c,n,o:2][C,c,n,o:3]</rxn>',<br>'reactants': ['O=C(O)C1Cc2ccc(Br)cc2C1',<br>'Cn1ncc(B2OC(C)(C)C(C)(C)O2)n1'],<br>'product': 'Cn1ncc(-c2ccc3c(c2)CC(C(=O)O)C3)n1'}<br>],<br>'building_blocks':<br>[<br>'<bb>NCCOCc1ccccc1Cl</bb>',<br>'<bb>O=C(O)C1Cc2ccc(Br)cc2C1</bb>',<br>'<bb>Cn1ncc(B2OC(C)(C)C(C)(C)O2)n1</bb>'<br>]<br>}"

Figure S1: Instruction, input, and output from the SynLlama model's inference on example SMILES string from Fig.1d. During data generation, all instructions remain the same, and the input-output pairs are generated within the training synthesizable chemical space. We enforce the JSON format in the output for our post processing algorithms. The output JSON has two parts: reactions and building blocks. In 'reactions', a series of reaction steps are generated, where the product of the next reaction serves as the reactant for the previous one. In 'building blocks', BBs are selected from the 'reaction' section and compiled into a list.

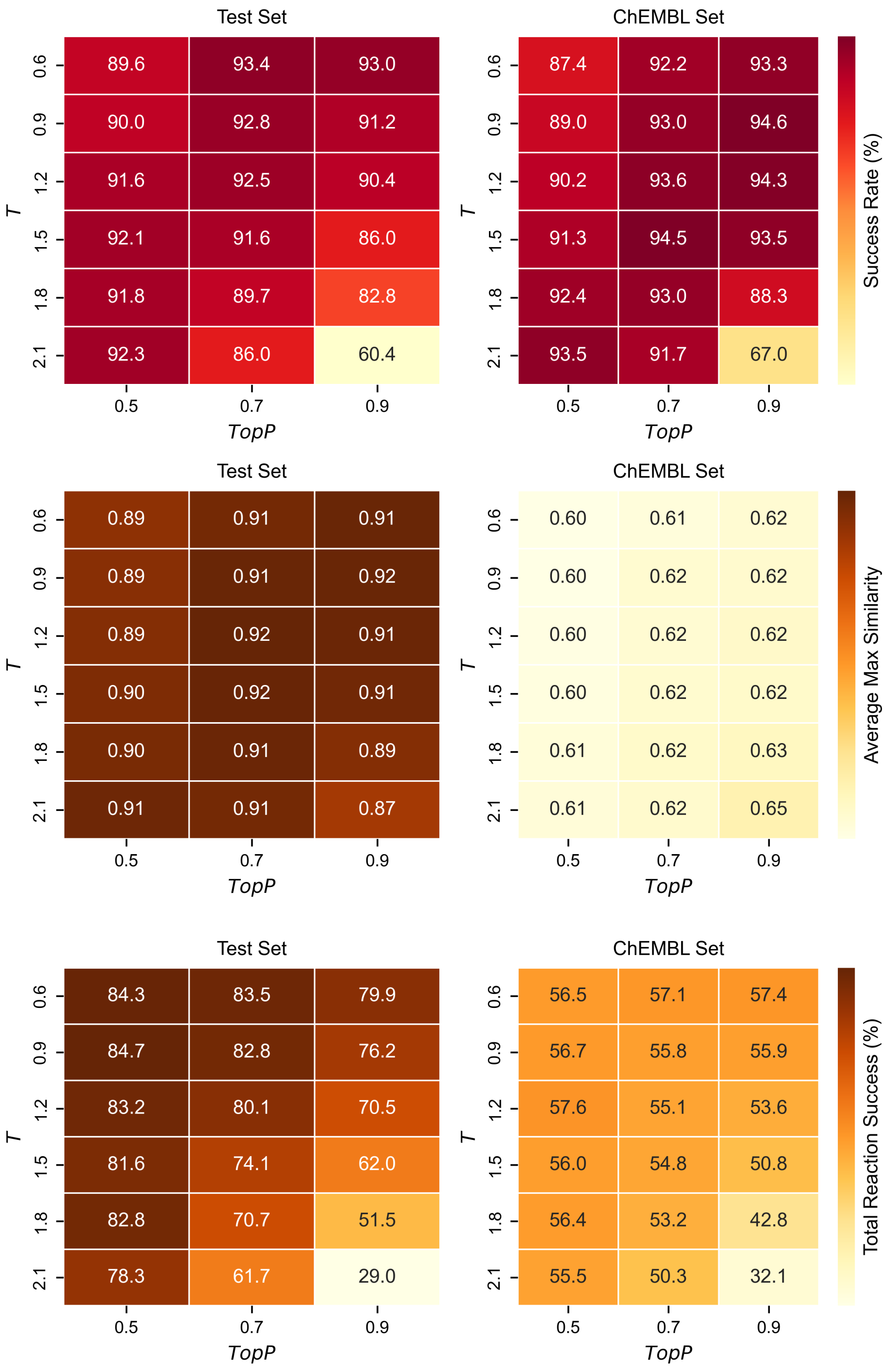

Figure S2: Reconstruction algorithm and SynLlama raw output benchmarks for SynLlama inferences on the Testing and ChEMBL sets under various temperature and top-p combinations. The first row represents the success rate of the Enamine reconstruction algorithm based on SynLlama inference outputs. The second row represents the average maximum Tanimoto similarity between the target and analogs generated via the reconstruction algorithm based on 4096-bit Morgan fingerprints. The last row represents the percentage of SynLlama raw outputs that can directly represent a retrosynthetic path for the input molecule without downstream processing with the reconstruction algorithm.

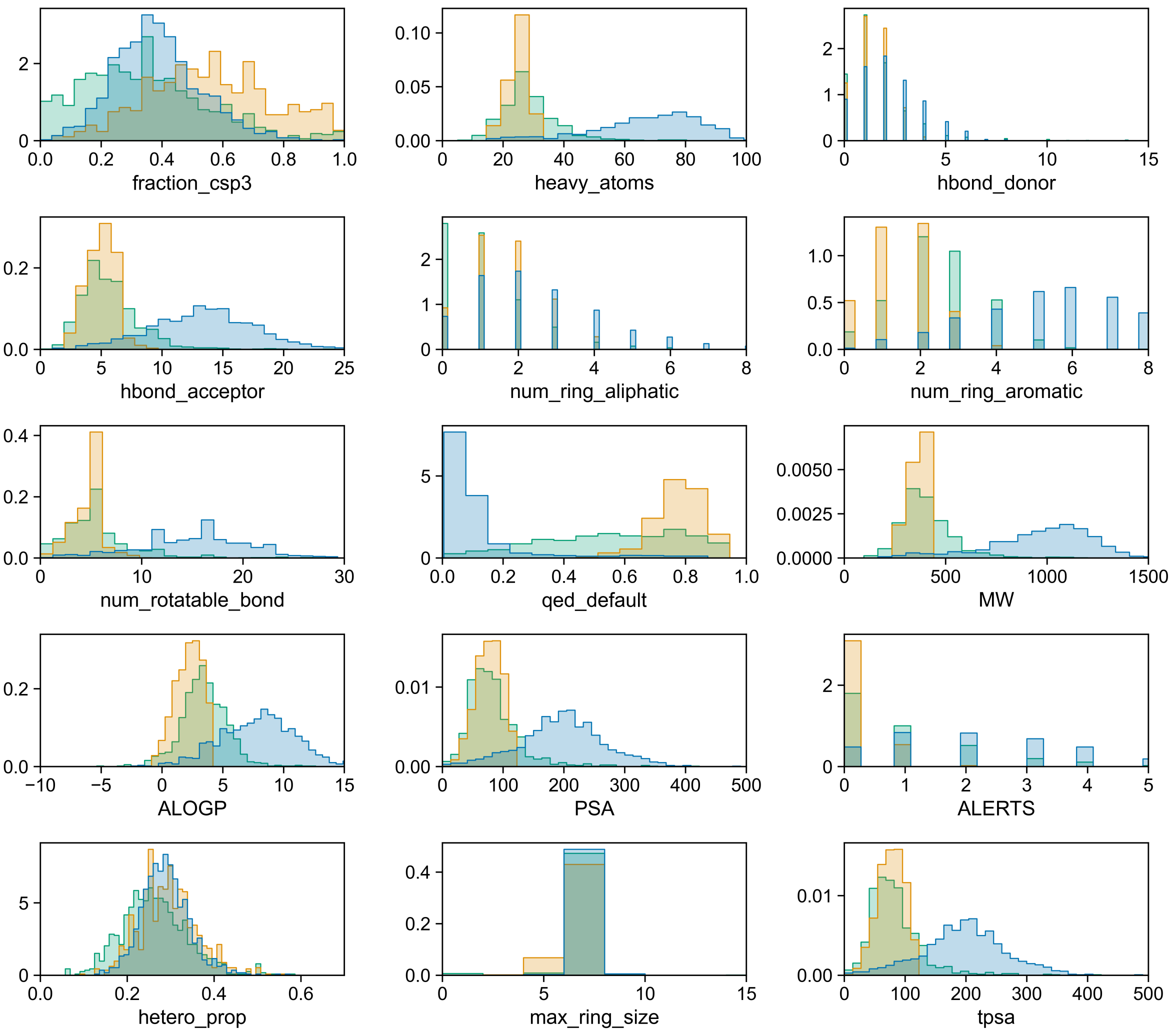

Figure S3: Drug-related property distributions between product molecules from normal training data (blue), Enamine Diversity Set(orange), and ChEMBL molecules (green).

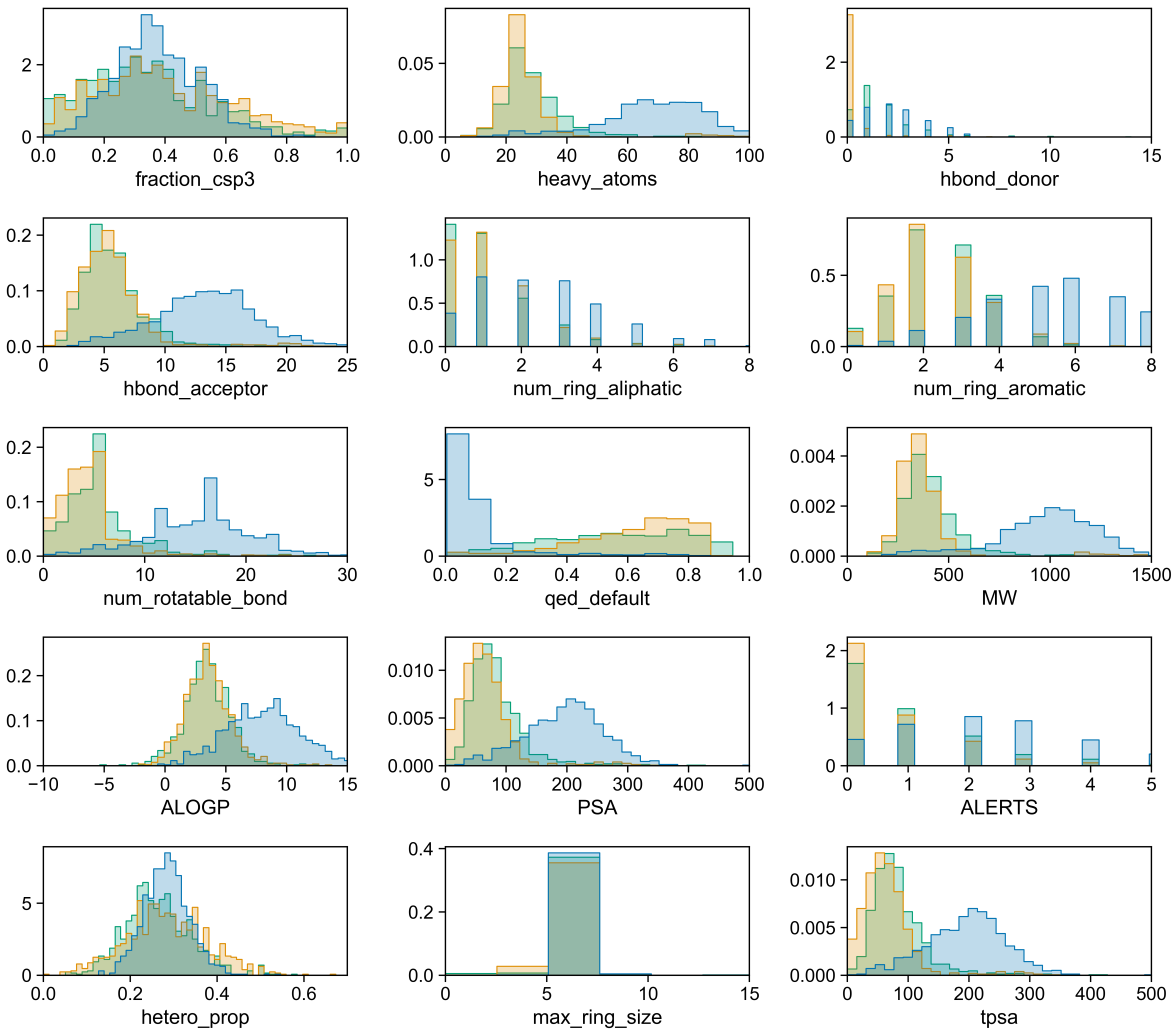

Figure S4: Drug-related property distributions between product molecules from normal training data (blue), product molecules from training data constrained on druglike properties (orange), and ChEMBL molecules (green). The generated product molecules under the constraint of druglike properties display similar distribution as ChEMBL molecules. The product molecules from normal training scheme occupies a very different chemical space with more larger molecules.

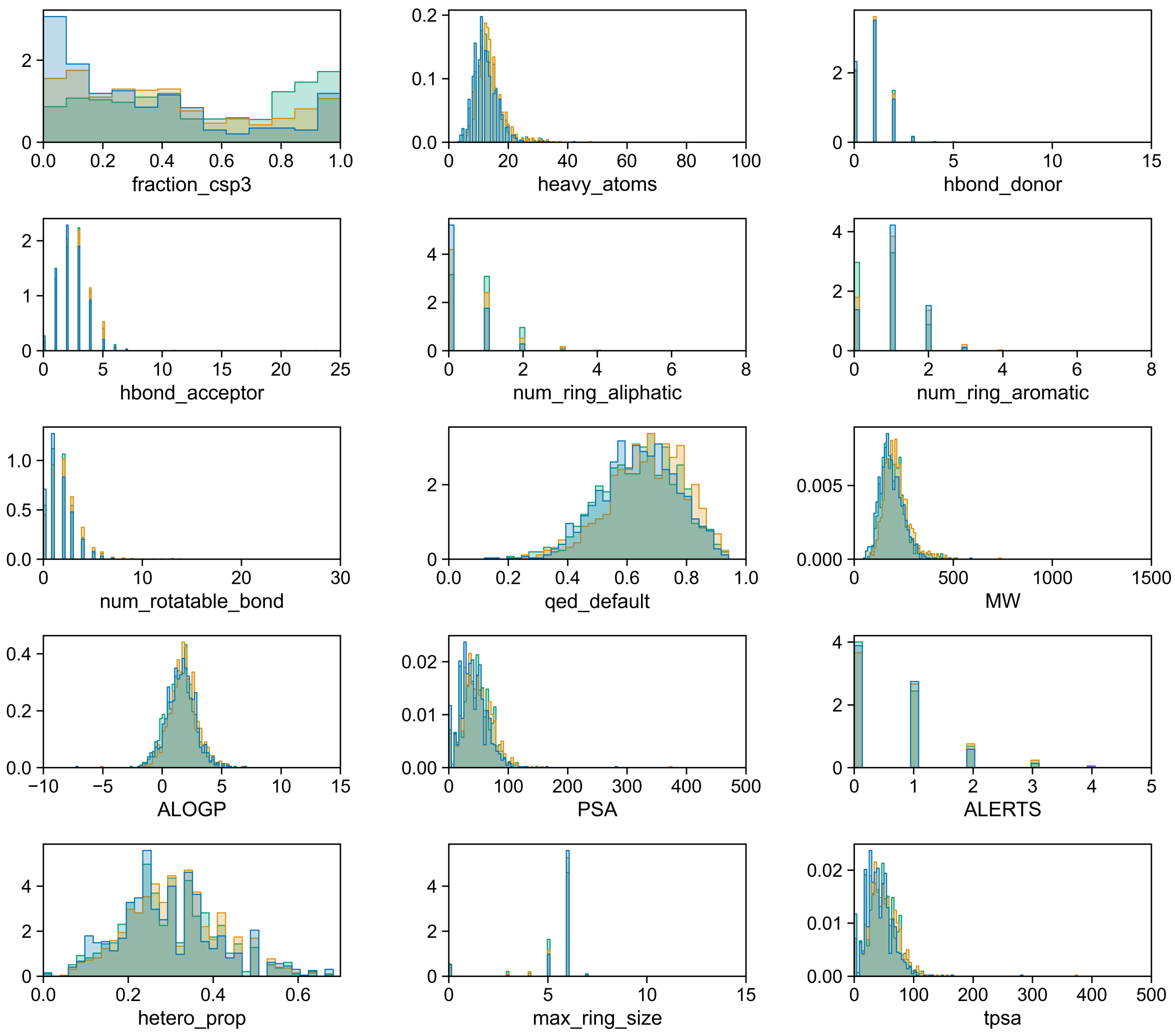

Figure S5: Drug-related property distributions between training building blocks (blue), testing building blocks (orange), and Molport building blocks (green). The three building blocks show very similar distribution in all categories.

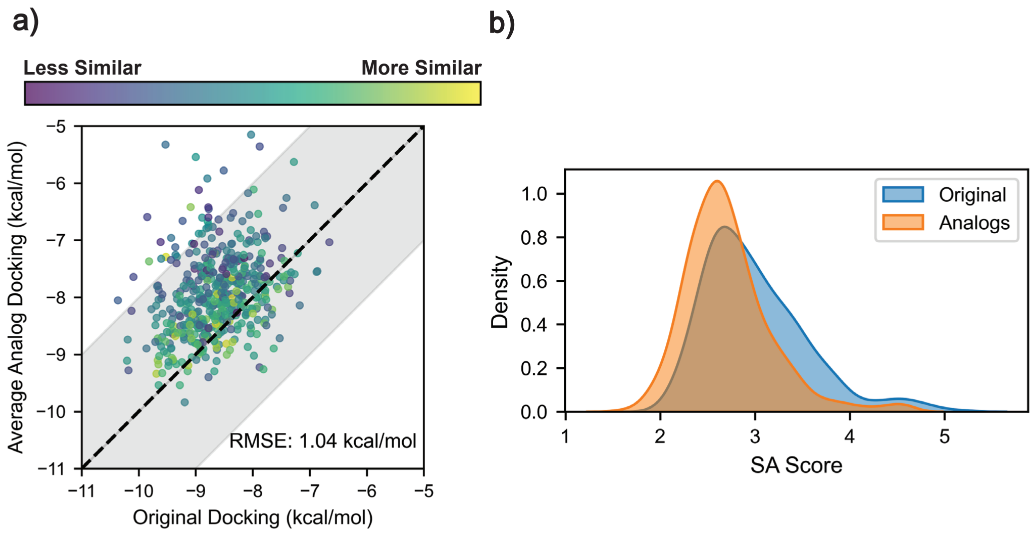

Figure S6: Docking score and SA score distribution between 500 iMiner-generated molecules and proposed analogs from SynLlama model trained on RXN 1. (a) Correlation plot comparing docking scores of 500 iMiner-generated molecules and the average docking scores of ten most similar analogs for each iMiner-generated molecule. Each data point is color-coded by the average Morgan fingerprint similarity computed between the iMiner target molecules and their corresponding analogs. The shaded area is the energy uncertainty range of $\pm 2kcal/mol$, which is typical for AutoDock Vina scores[15]. (b) SA score distribution of iMiner molecules and SynLlama-proposed analogs.

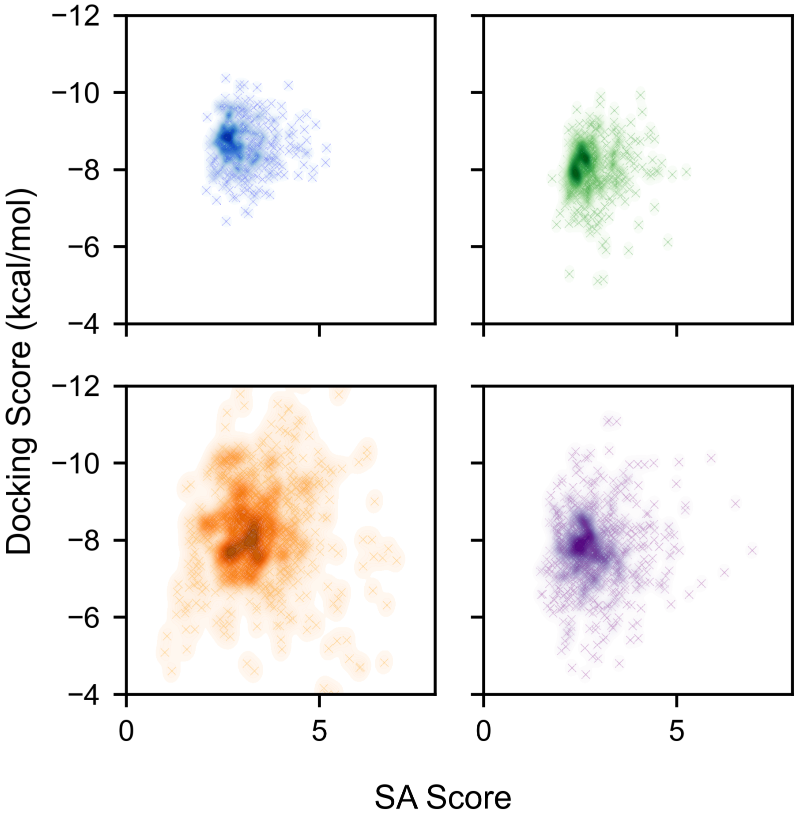

Figure S7: Kernel density estimations of docking scores and SA score for target molecules and SynLlama-generated analogs. Blue: iMiner targets. Green: iMiner analogs. Orange: Pocket2Mol targets. Purple: Pocket2Mol analogs.

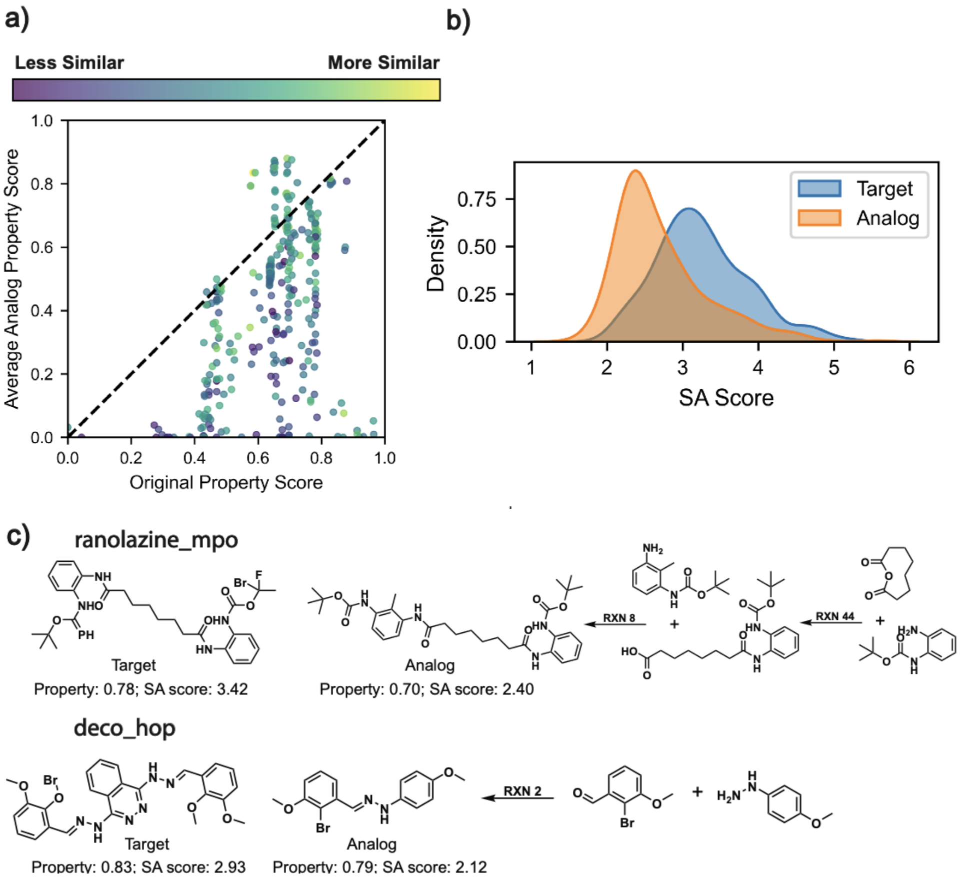

Figure S8: Oracle score and SA score distribution between 500 ASKCOS unsynthesizable molecules and proposed analogs from SynLlama model trained on RXN 2. (a) Correlation plot comparing property scores of 500 ASKCOS unsynthesizable molecules and the average docking scores of ten most similar analogs for each molecule. Each data point is color-coded by the average Morgan fingerprint similarity computed between the ASKCOS unsynthesizable molecules and their corresponding analogs. (b) SA score distribution of ASKCOS unsynthesizable molecules and SynLlama-proposed analogs. (c) Property and SA scores for example target-analog pairs along with the predicted analog synthetic pathways for two optimization targets: Ranolazine MPO and Deco Hop.

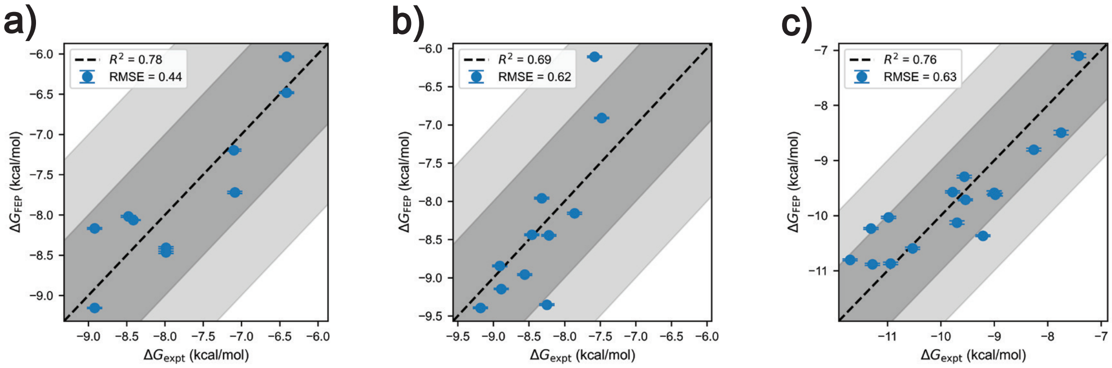

Figure S9: FEP benchmarking on all three protein systems. Correlation plots between ΔG extracted experimental IC50 values and ΔG calculated from FEP for (a) SARS-CoV-2 Mpro[34], (b) Thrombin[35], and (c) TYK2[36,37]. The correlations across all three systems have RMSE < 1 kcal/mol, indicating the reliability of FEP calculations.

References

[1] Dubey, A. et al. The llama 3 herd of models (2024). URL http://arxiv.org/abs/2407.21783. 2407.21783[cs].

[2] Lian, W. axolotl. url https://github.com/axolotl-ai-cloud/axolotl/tree/main. URL https://github.com/axolotl-ai-cloud/axolotl/tree/main.

[3] Hu, E. J. et al. LoRA: Low-rank adaptation of large language models. In International Conference on Learning Representations (2022). URL https://openreview.net/forum?id=nZeVKeeFYf9.

[4] Dao, T. FlashAttention-2: Faster attention with better parallelism and work partitioning. In International Conference on Learning Representations (ICLR) (2024).

[5] Kingma, D. P. & Ba, J. Adam: A method for stochastic optimization. In Bengio, Y. & LeCun, Y. (eds.) 3rd International Conference on Learning Representations, ICLR 2015, San Diego, CA, USA, May 7- (2015). URL http://arxiv.org/abs/1412.6980.

[6] Spärck Jones, K. A statistical interpretation of term specificity and its application in retrieval. Journal of Documentation **28**, 11–21 (1972).

[7] Gao, W., Mercado, R. & Coley, C. W. Amortized Tree Generation for Bottom-up Synthesis Planning and Synthesizable Molecular Design (2022). URL http://arxiv.org/abs/2110.06389. ArXiv:2110.06389.

[8] Morgan, H. L. The generation of a unique machine description for chemical structures—a technique developed at chemical abstracts service. Journal of Chemical Documentation **5**, 107–113 (1965).

[9] Rogers, D. J. & Tanimoto, T. T. A Computer Program for Classifying Plants: The computer is programmed to simulate the taxonomic process of comparing each case with every other case. Science **132**, 1115–1118 (1960). URL https://www.science.org/doi/10.1126/science.132.3434.1115.

[10] Luo, S. et al. Projecting Molecules into Synthesizable Chemical Spaces (2024). URL http://arxiv.org/abs/2406.04628. ArXiv:2406.04628.

[11] Gao, W., Luo, S. & Coley, C. W. Generative Artificial Intelligence for Navigating Synthesizable Chemical Space (2024). URL http://arxiv.org/abs/2410.03494. ArXiv:2410.03494.

[12] Huang, K. et al. Therapeutics data commons: Machine learning datasets and tasks for drug discovery and development. Proceedings of Neural Information Processing Systems, NeurIPS Datasets and Benchmarks (2021).

[13] Li, J. et al. Mining for potent inhibitors through artificial intelligence and physics: A unified methodology for ligand based and structure based drug design. Journal of Chemical Information and Modeling (2024). URL https://doi.org/10.1021/acs.jcim.4c00634.

[14] Krenn, M., Häse, F., Nigam, A., Friederich, P. & Aspuru-Guzik, A. Self-referencing embedded strings (selfies): A 100% robust molecular string representation. Mach. Learn.: Sci. Tech. **1**, 045024 (2020).

[15] Trott, O. & Olson, A. J. AutoDock Vina: Improving the speed and accuracy of docking with a new scoring function, efficient optimization, and multithreading. J. Comp. Chem. **31**, 455–461 (2010). URL https://doi.org/10.1002/jcc.21334.

[16] Zhang, C.-H. et al. Potent noncovalent inhibitors of the main protease of sars-cov-2 from molecular sculpting of the drug perampanel guided by free energy perturbation calculations. ACS Cent. Sci. **7**, 467–475 (2021).

[17] Berman, H. et al. The protein data bank. Nucleic Acids Research **28**, 235–242 (2000).

[18] Eastman, P. et al. Openmm 4: A reusable, extensible, hardware independent library for high performance molecular simulation. Journal of Chemical Theory and Computation **9**, 461–469 (2013).

[19] Pocket2mol. https://github.com/pengxingang/Pocket2Mol. Accessed on Jun 24, 2025.

[20] Schrodinger-fep-benchmark. https://github.com/schrodinger/public_binding_free_energy_benchmark. Accessed on June 24, 2025.

[21] Tu, Z. et al. ASKCOS: Open-Source, Data-Driven Synthesis Planning. Accounts of Chemical Research **58**, 1764–1775 (2025). URL https://pubs.acs.org/doi/10.1021/acs.accounts.5c00155.

[22] Case, D. A. et al. Amber22 (2022).

[23] Maier, J. A. et al. ff14sb: improving the accuracy of protein side chain and backbone parameters from ff99sb. Journal of chemical theory and computation **11**, 3696–3713 (2015).

[24] Boothroyd, S. et al. Development and benchmarking of open force field 2.0.0: The sage small molecule force field. J. Chem. Theo. Comp. **19**, 3251–3275 (2023). URL https://doi.org/10.1021/acs.jctc.3c00039.

[25] Pdbfixer. https://github.com/openmm/pdbfixer. Accessed on Oct 29, 2024.

[26] Eastman, P. et al. Openmm 8: Molecular dynamics simulation with machine learning potentials. The Journal of Physical Chemistry B **128**, 109–116 (2024). URL https://doi.org/10.1021/acs.jpcb.3c06662. PMID: 38154096, https://doi.org/10.1021/acs.jpcb.3c06662.

[27] Chen, W. et al. Accurate calculation of relative binding free energies between ligands with different net charges. Journal of chemical theory and computation **14**, 6346–6358 (2018).

[28] Tsai, H.-C. et al. Amber free energy tools: a new framework for the design of optimized alchemical transformation pathways. Journal of chemical theory and computation **19**, 640–658 (2023).

[29] Ries, B. et al. Kartograf: A geometrically accurate atom mapper for hybrid-topology relative free energy calculations. Journal of Chemical Theory and Computation **20**, 1862–1877 (2024).

[30] Lee, T.-S., Tsai, H.-C., Ganguly, A. & York, D. M. Aces: optimized alchemically enhanced sampling. Journal of chemical theory and computation **19**, 472–487 (2023).

[31] Hopkins, C. W., Le Grand, S., Walker, R. C. & Roitberg, A. E. Long-time-step molecular dynamics through hydrogen mass repartitioning. Journal of chemical theory and computation **11**, 1864–1874 (2015).

[32] Wu, Z. et al. alchemlyb: the simple alchemistry library. Journal of Open Source Software **9**, 6934 (2024). URL `https://doi.org/10.21105/joss.06934`.

[33] Xu, H. Optimal measurement network of pairwise differences. Journal of Chemical Information and Modeling **59**, 4720–4728 (2019).

[34] Kneller, D. W. et al. Structural, electronic, and electrostatic determinants for inhibitor binding to subsites s1 and s2 in sars-cov-2 main protease. Journal of Medicinal Chemistry **64**, 17366–17383 (2021). URL `https://doi.org/10.1021/acs.jmedchem.1c01475`. PMID: 34705466, `https://doi.org/10.1021/acs.jmedchem.1c01475`.

[35] Baum, B. et al. More than a simple lipophilic contact: a detailed thermodynamic analysis of nonbasic residues in the s1 pocket of thrombin. Journal of molecular biology **390**, 56–69 (2009).

[36] Liang, J. et al. Lead identification of novel and selective TYK2 inhibitors. European Journal of Medicinal Chemistry **67**, 175–187 (2013). URL `https://linkinghub.elsevier.com/retrieve/pii/S0223523413002304`.

[37] Liang, J. et al. Lead Optimization of a 4-Aminopyridine Benzamide Scaffold To Identify Potent, Selective, and Orally Bioavailable TYK2 Inhibitors. Journal of Medicinal Chemistry **56**, 4521–4536 (2013). URL `https://doi.org/10.1021/jm400266t`. Publisher: American Chemical Society.
